# The Wdr1-LIMK-Cofilin Axis Controls B Cell Antigen Receptor-Induced Actin Remodeling and Signaling at the Immune Synapse

**DOI:** 10.3389/fcell.2021.649433

**Published:** 2021-04-13

**Authors:** Madison Bolger-Munro, Kate Choi, Faith Cheung, Yi Tian Liu, May Dang-Lawson, Nikola Deretic, Connor Keane, Michael R. Gold

**Affiliations:** Department of Microbiology & Immunology and Life Sciences Institute, University of British Columbia, Vancouver, BC, Canada

**Keywords:** B cell, actin, immune synapse, cell spreading, cofilin, WDR1 (AIP1), LIM domain kinase, B cell receptor (BCR)

## Abstract

When B cells encounter membrane-bound antigens, the formation and coalescence of B cell antigen receptor (BCR) microclusters amplifies BCR signaling. The ability of B cells to probe the surface of antigen-presenting cells (APCs) and respond to APC-bound antigens requires remodeling of the actin cytoskeleton. Initial BCR signaling stimulates actin-related protein (Arp) 2/3 complex-dependent actin polymerization, which drives B cell spreading as well as the centripetal movement and coalescence of BCR microclusters at the B cell-APC synapse. Sustained actin polymerization depends on concomitant actin filament depolymerization, which enables the recycling of actin monomers and Arp2/3 complexes. Cofilin-mediated severing of actin filaments is a rate-limiting step in the morphological changes that occur during immune synapse formation. Hence, regulators of cofilin activity such as WD repeat-containing protein 1 (Wdr1), LIM domain kinase (LIMK), and coactosin-like 1 (Cotl1) may also be essential for actin-dependent processes in B cells. Wdr1 enhances cofilin-mediated actin disassembly. Conversely, Cotl1 competes with cofilin for binding to actin and LIMK phosphorylates cofilin and prevents it from binding to actin filaments. We now show that Wdr1 and LIMK have distinct roles in BCR-induced assembly of the peripheral actin structures that drive B cell spreading, and that cofilin, Wdr1, and LIMK all contribute to the actin-dependent amplification of BCR signaling at the immune synapse. Depleting Cotl1 had no effect on these processes. Thus, the Wdr1-LIMK-cofilin axis is critical for BCR-induced actin remodeling and for B cell responses to APC-bound antigens.

## Introduction

Signaling by the B cell antigen receptor (BCR) initiates the B cell activation process. Activated B cells provide protective immunity by producing antibodies, secreting cytokines, and presenting antigens (Ags) to T cells, but can also contribute to autoimmunity (Conley et al., 2009; Shen and Fillatreau, 2015; Cashman et al., 2019; Cyster and Allen, 2019; Meffre and O'Connor, 2019). Within lymphoid organs, Ag-presenting cells (APCs) such as follicular dendritic cells, dendritic cells, and subcapsular sinus macrophages increase the efficiency of B cell activation by capturing Ags and concentrating them on their surface (Batista and Harwood, 2009; Cyster, 2010; Heesters et al., 2016). The interaction of B cells with Ags that are mobile within a membrane initiates the reorganization of B-cell membrane proteins into an immune synapse, thereby enhancing both the signal transduction and Ag internalization functions of the BCR (Harwood and Batista, 2011; Song et al., 2014; Kuokkanen et al., 2015). Ag-bound BCRs rapidly form microclusters, which nucleate protein complexes that activate the signaling pathways controlled by phospholipase C, phosphoinositide 3-kinase, and the Ras, Rac, Cdc42, and Rap1 GTPases (Treanor et al., 2009; Packard and Cambier, 2013; Abraham et al., 2016). The centripetal movement and coalescence of BCR microclusters further amplifies microcluster-based BCR signaling, increasing the probability that the magnitude of BCR signaling exceeds the threshold for B cell activation (Bolger-Munro et al., 2019). Ultimately, BCR-Ag microclusters coalesce into a central supramolecular activation cluster (cSMAC) (Fleire et al., 2006). cSMAC formation may enhance BCR-mediated Ag internalization, which is required for B cells to present Ags to T cells and elicit second signals for B cell activation (Yuseff et al., 2013; Nowosad et al., 2016). Hence, elucidating the mechanisms that drive immune synapse formation is critical for understanding how APC-bound Ags activate B cells.

Dynamic remodeling of the actin cytoskeleton is required for immune synapse formation (Harwood and Batista, 2011; Song et al., 2014). We identified a critical role for actin-related protein 2/3 (Arp2/3) complex-nucleated actin polymerization in multiple aspects of this process (Bolger-Munro et al., 2019). The Arp2/3 complex binds to existing actin filaments and nucleates the formation of new filaments that grow at a 70° angle to the mother filament (Goley and Welch, 2006). This branched actin assembly creates a dendritic actin network that can exert outward force on the plasma membrane and drive the formation of broad lamellipodial protrusions (Mogilner and Oster, 1996). When B cells contact Ag-bearing surfaces, BCR-induced actin polymerization at the cell periphery allows B cells to extend membrane protrusions in order to scan more of the surface and encounter more Ag (Bolger-Munro et al., 2019). When Ags are mobile within a membrane, the B cell then retracts these membrane protrusions (Fleire et al., 2006), with BCR microclusters undergoing centripetal movement and coalescing into a cSMAC. We showed that these processes are driven by Arp2/3 complex-dependent actin retrograde flow (i.e., away from the cell periphery and toward the center of the cell-cell contact site) within the peripheral actin network (Bolger-Munro et al., 2019). Actin retrograde flow is a consequence of the elastic resistance of the cell membrane exerting an opposing inward force when actin polymerization at the cell membrane exerts outwards forces (Ponti et al., 2004). When the Arp2/3 complex is inhibited or depleted, the retraction of membrane protrusions is impaired, the centripetal movement of BCR microclusters is greatly reduced, and cSMAC formation is inhibited (Bolger-Munro et al., 2019). Importantly, this results in decreased microcluster-based BCR signaling and impaired B cell activation in response to APC-bound Ags (Bolger-Munro et al., 2019). Consistent with these findings, human mutations in the Arpc1B component of the Arp2/3 complex, or in activators of the Arp2/3 complex such as Wiskott-Aldrich Syndrome protein (WASp) and the Hem1 component of the WAVE regulatory complex, result in B cell dysfunction (Kahr et al., 2017; Kuijpers et al., 2017; Brigida et al., 2018; Candotti, 2018; Volpi et al., 2019; Cook et al., 2020; Sprenkeler et al., 2020).

Actin network assembly that is nucleated by the Arp2/3 complex occurs concurrently with the actions of actin disassembly factors such as cofilin, destrin (also known as actin-depolymerizing factor [ADF]), and gelsolin, which bind to and sever actin filaments (Ono, 2003; Bernstein and Bamburg, 2010; Shishkin et al., 2016). Released filament segments undergo depolymerization and the resulting actin monomers can be loaded with ATP and used for new actin polymerization (Kadzik et al., 2020). Actin network disassembly also releases Arp2/3 complexes from branch points, allowing them to be recycled and initiate the formation of new branches. In lamellipodia, the actions of the Arp2/3 complex and cofilin are tightly coupled but spatially separated (Carlier et al., 1997; Svitkina and Borisy, 1999). Cofilin severs older portions of actin filaments, which are further from the cell membrane, and in which the ATP bound to constituent actin monomers has been hydrolyzed to ADP (Pollard and Borisy, 2003; Bernstein and Bamburg, 2010). At the same time, Arp2/3 complex-dependent actin polymerization occurs primarily at the plasma membrane where membrane-bound activators of the Arp2/3 complex, such as WASp and WAVE, bind ATP-loaded actin monomers and deliver them directly to the Arp2/3 complex (Bieling et al., 2018; Mullins et al., 2018). This balanced actin polymerization and depolymerization is termed treadmilling (Carlier and Shekhar, 2017).

In addition to fueling actin polymerization, actin severing is essential for the remodeling of actin networks. Cofilin is a major actin-severing protein in murine splenic B cells (Freeman et al., 2011). We have previously shown that cofilin-mediated actin severing is required for B cell spreading as well as APC-induced microcluster formation and BCR signaling (Freeman et al., 2011). This suggests that proteins that regulate cofilin-mediated actin severing may also be important regulators of actin dynamics and immune synapse formation in B cells.

Cofilin activity is regulated by phosphorylation on serine 3 (S3), which prevents cofilin from binding to actin filaments (Bravo-Cordero et al., 2013). Dephosphorylation of cofilin on S3 causes a conformational change that allows cofilin to bind actin filaments and carry out its severing activity. The major phosphatases that dephosphorylate cofilin belong to the Slingshot (SSH) family (Niwa et al., 2002; Kanellos and Frame, 2016), although other widely expressed phosphatases such as PP1 and PP2A may also perform this function (Ambach et al., 2000; Ohashi, 2015). LIM domain kinase (LIMK) 1 and 2 are widely-expressed kinases that phosphorylate cofilin on S3 (Ohashi, 2015). The LIMKs are activated via phosphorylation by Rho-associated protein kinase (ROCK), a downstream target of the Rho GTPase, or by p21-activated kinase (PAK), an effector of the Rac and Cdc42 GTPases (Scott and Olson, 2007; Prunier et al., 2017). The Rho-ROCK-LIMK pathway modulates immune synapse formation and function in T cells (Thauland et al., 2017). Inhibiting cofilin activity by expressing constitutively active ROCK, or by depleting cofilin with siRNA, results in smaller immune synapses (i.e., less spreading on the APC surface) and decreased TCR-induced Ca^2+^ flux. Conversely, inhibiting the activity of ROCK or LIMK, which increases the amount of active cofilin, results in larger immune synapses, and increased Ca^2+^ flux. The role of LIMK in BCR-induced actin remodeling and B cell responses to APCs has not been investigated.

In addition to proteins that regulate the phosphorylation of cofilin on S3, a number of other proteins modulate cofilin-mediated actin severing. In this study we examined the role of WD repeat-containing protein 1 (Wdr1; also known as actin-interacting protein 1 [Aip1]) and coactosin-like protein 1 (Cotl1) in B cell spreading and responses to APCs. Wdr1 binds to cofilin-decorated actin filaments and increases the rate of cofilin-mediated actin severing (Rodal et al., 1999; Nadkarni and Brieher, 2014; Chen et al., 2015; Nomura et al., 2016; Ono, 2018). *In vitro*, actin filaments are stabilized when they are saturated with cofilin. However, Wdr1 optimizes the spacing of cofilin on actin filaments so that it is favorable for severing, which occurs when strain builds up at the boundaries between cofilin-decorated and bare regions (Elam et al., 2013; Gressin et al., 2015; Tanaka et al., 2018). Wdr1 creates cofilin-bare regions of actin filaments by competing with cofilin for binding to polymerized actin (Nadkarni and Brieher, 2014; Chen et al., 2015) or by inducing a conformational change in the actin binding site of cofilin that reduces its affinity for actin filaments (Aggeli et al., 2014). In addition, Wdr1-cofilin interactions at the boundary between cofilin-decorated and cofilin-bare regions of the filament promote severing at that site (Hayakawa et al., 2019). In yeast and in mammalian cell extracts the absence of Wdr1 results in reduced actin filament turnover, accumulation of actin filaments, and depletion of the actin monomer pool (Okreglak and Drubin, 2010; Nadkarni and Brieher, 2014). Importantly, loss-of-function mutations in human *Wdr1* are associated with an immunodeficiency syndrome characterized by defective motility of myeloid cells, aberrant T cell activation, and impaired B cell development (Pfajfer et al., 2018).

Cotl1 is a member of the cofilin/ADF superfamily (Shishkin et al., 2016) that is structurally homologous to cofilin and binds actin filaments with high affinity (Provost et al., 2001). *In vitro*, Cotl1 competes with cofilin for binding to actin filaments. However, in contrast to Wdr1 it stabilizes actin filaments and attenuates cofilin-mediated severing (Provost et al., 2001; Kim et al., 2014). In T cells, Cotl1 is recruited to the immune synapse where it promotes the formation of lamellipodial protrusions (Kim et al., 2014) but its function in B cells has not been studied.

Because cofilin initiates actin remodeling and fuels Arp2/3 complex-nucleated actin polymerization, we tested the hypothesis that the Wdr1-LIMK-cofilin axis and Cotl1 regulate B cell spreading, APC-induced BCR signaling, and cSMAC formation at the immune synapse.

## Materials and Methods

### B Cells

The A20 murine IgG^+^ B cell line was obtained from ATCC (#TIB-208). A20 D1.3 B cells, which express a transfected hen egg lysozyme (HEL)-specific BCR, were from F. Batista (Ragon Institute, Cambridge, MA) (Batista and Neuberger, 1998). Both cell lines were confirmed to be mycoplasma-negative and were cultured in RPMI-1640 supplemented with 5% heat-inactivated fetal calf serum (FCS), 2 mM glutamine, 1 mM pyruvate, 50 μM 2-mercaptoethanol, 50 U/mL penicillin, and 50 μg/mL streptomycin. A20 and A20 D1.3 B cells (2 × 10^6^) were transiently transfected with 2 μg siRNA using AMAXA Cell Line Nucleofector Kit V (Lonza, #VCA-1003) or the Ingenio Electroporation Kit (Mirus, #MIR 50118). The siRNAs used were control non-targeting siRNA (ON-TARGETplus Non-Targeting Pool, Dharmacon, #D-00810-01-05), cofilin-1 siRNA (Dharmacon, #L-058638-01-0005), Wdr1 siRNA (SMARTpool ON-TARGETplus, Dharmacon, #L-047667-01-005), and Cotl1 siRNA (ON-TARGETplus, Dharmacon, #L-042151-01-005). Transfected A20 and A20 D1.3 B cells were cultured for 48 h before being used for experiments. siRNA-mediated decreases in protein levels were assessed by immunoblotting (see below). Murine primary B cells were isolated from the spleens of 8-−12-week old C57BL/6J mice (Jackson Laboratories, #000664) or MD4 mice (Jackson Laboratories, #002595) of either sex using a negative selection B cell isolation kit (Stemcell Technologies, #19854A). Animal protocols were approved by the University of British Columbia Animal Care Committee. Where indicated, *ex vivo* primary B cells, A20 B cells, or A20 D1.3 B cells were pre-treated for 1 h with the LIMK inhibitor, LIMKi3 (Tocris, #4745) (Ross-Macdonald et al., 2008; Scott et al., 2010), or with an equal volume of DMSO.

### Analysis of Cell Surface BCR Levels and Filamentous Actin Content by Flow Cytometry

A20 or A20 D1.3 B cells that had been transfected with siRNA, or treated with either DMSO or LIMKi3, were fixed with 4% paraformaldehyde (PFA) in PBS for 10 min at room temperature and then resuspended in ice-cold FACS buffer (PBS, 2% FCS, 0.02% NaN_3_). Fc receptors were blocked by adding 25 μg/mL of the 2.4G2 anti-mouse CD16/CD32 monoclonal antibody for 5 min on ice. To assess cell surface BCR levels, the cells were stained on ice for 30 min with goat anti-mouse IgG-Alexa Fluor 647 (Invitrogen, #A21236, 1:200) or with rat anti-mouse IgM-FITC (eBiosciences, #11-5890-85, 1:200) to detect the D1.3 BCR. Intracellular filamentous actin (F-actin) was detected by permeabilizing the PFA-fixed cells with 0.2% Triton X-100 for 5 min on ice and then staining with rhodamine-phalloidin (Invitrogen, #R415, 1:100) for 30 min on ice. Flow cytometry was performed using an LSRII-561 cytometer (Becton Dickinson Biosciences) and data were analyzed using FlowJo software (Treestar Inc.), gating on single intact cells using forward and side scatter.

### BCR Signaling in Response to Soluble Anti-Ig

B cells were resuspended to 2 × 10^7^ cells/mL in modified HEPES-suffered saline (mHBS; 25 mM HEPES, pH 7.2, 125 mM NaCl, 5 mM KCl, 1 mM CaCl_2_, 1 mM Na_2_HPO_4_, 1 mg/mL glucose, 2 mM glutamine, 1 mM pyruvate, 50 μM 2-mercaptoethanol). The cells (3 × 10^6^ in 150 μL) were then stimulated with 20 μg/mL goat anti-mouse IgG (Jackson ImmunoResearch, #115-005-008) or goat anti-mouse IgM (Jackson ImmunoResearch, ##115-005-020) for the indicated times at 37°C. Reactions were stopped by adding cold PBS with 1 mM Na_3_VO_4_. The cells were then pelleted for 5 min at 640 RCF at 4°C and lysed in RIPA buffer (30 mM Tris-HCl, pH 7.4, 150 mM NaCl, 1% Igepal (Sigma-Aldrich), 0.5% sodium deoxycholate, 0.1% SDS, 2 mM EDTA) with protease and phosphatase inhibitors (1 mM phenylmethylsulfonyl fluoride, 10 μg/mL leupeptin, 1 μg/mL aprotinin, 1 μg/mL pepstatin A, 10 μg/mL soybean trypsin inhibitor, 25 mM β-glycerophosphate, 1 mM Na_3_MoO_4_, 1 mM Na_3_VO_4_). Protein concentrations were determined using the bicinchoninic acid assay (Thermo Fisher, #23225). Cell extracts were analyzed by immunoblotting.

### Immunoblotting

Cell extracts were separated on 12% SDS-PAGE gels and transferred to nitrocellulose membranes, which were blocked with 5% milk powder in Tris-buffered saline (10 mM Tris-HCl, pH 8, 150 mM NaCl). The membranes were incubated overnight at 4°C with mouse anti-Wdr1 (Santa Cruz, #sc-393159; 1:500), sheep anti-Cotl1 (R&D Systems, #AF7865; 1:500), rabbit anti-CD79a (1:5,000) (Gold et al., 1991), or the following rabbit antibodies from Cell Signaling Technologies: phosphorylated CD79a (pCD79a; #5173; 1:1,000), phosphorylated Erk (pERK; #9101; 1:1,000), Erk (#9102, 1:1,000), phosphorylated cofilin (p-cofilin; #3313; 1:1,000), or cofilin (#3318; 1:1,000). Immunoreactive bands were visualized using horseradish peroxidase-conjugated goat anti-rabbit IgG (Bio-Rad, #170-6515; 1:3,000), mouse Igκ-binding protein (Santa Cruz, #sc-516102, 1:2,000), or donkey anti-sheep IgG (R&D Systems, #HAF016, 1:1,000), followed by ECL detection (Azure Biosystems, #AC2010). All antibodies were diluted in Tris-buffered saline. Blots were quantified and imaged using a Li-Cor C-DiGit imaging system.

### Ca^2+^ Flux Assays

A20 or A20 D1.3 B cells that had been transfected with siRNA, or treated with DMSO or LIMKi3, were washed twice with Hanks' Balanced Salt Solution (HBSS) containing 10 mM HEPES and resuspended to 10^7^ per mL before adding 2 μM Fura Red (Invitrogen, #F3021), 1 μM Fluo-4 (Invitrogen, #F14201), and 0.02% Pluronic F-127 (Invitrogen, #P3000MP). The cells were then incubated for 30 min at room temperature protected from light, washed with HBSS/10 mM HEPES/2% FCS, resuspended to 10^7^/mL, and incubated for an additional 20 min protected from light. Flow cytometry was performed using an LSRII-561 cytometer (Becton Dickinson Biosciences). For each sample, 1–3 × 10^6^ cells were pelleted and resuspended in 0.5 mL mHBS, with paired samples having similar number of cells. Samples were analyzed for 1 min to establish baseline values before adding either goat anti-mouse IgG (Jackson ImmunoResearch, #115-005-008, 20 μg/mL) for A20 B cells or goat anti-mouse IgM (Jackson ImmunoResearch, #115-005-020, 20 μg/mL) for A20 D1.3 B cells, and then analyzing the cells for an additional 5 min. Ionomycin (1 μM; Invitrogen, # I24222) was added to saturate the Ca^2+^-sensing dyes and the cells were analyzed for an additional 1 min. Data were analyzed using FlowJo software (Treestar Inc.), gating on single intact cells using forward and side scatter.

### Cell Area, Actin Organization, and Actin Dynamics in B Cells Spreading on Immobilized Anti-Ig

Glass coverslips were coated with 2.5 μg/cm^2^ goat anti-mouse IgG (Jackson ImmunoResearch, #115-005-008) and then blocked with 2% bovine serum albumin (BSA) in PBS, as described previously (Lin et al., 2008). A20 B cells were resuspended in mHBS, or in mHBS + 2% FCS (imaging medium) before adding 7.5 × 10^4^ cells (in 100 μL) to each coverslip. At the indicated times, the cells were fixed with 4% PFA for 10 min and then permeabilized with 0.2% Triton X-100 in PBS for 5 min at room temperature. F-actin was visualized by staining with rhodamine-conjugated phalloidin (Thermo Fisher, #R415, 1:400 in PBS + 2% BSA) for 30 min at room temperature. Coverslips were mounted onto slides using ProLong Diamond anti-fade reagent (Thermo Fisher, #P36965). Images of the B cell-coverslip interface were captured using a laser scanning confocal microscope (Leica Microsystems TCS SP5) with a 60X NA 1.4 oil objective lens. The cell area, as well as the percent of the cell area that was depleted of F-actin, was quantified from thresholded binary images using Fiji software (Schindelin et al., 2012). The outer face of the peripheral actin ring was used to define the cell edge and compute the total cell area. The inner face of the peripheral actin ring was used to delimit the central actin-depleted region of the cell and calculate its area.

For live-cell imaging at 37°C, A20 B cells were transfected with a plasmid encoding F-tractin-GFP (Johnson and Schell, 2009), or co-transfected with F-tractin-GFP and siRNAs, 48 h before being used for experiments. Cells (5 × 10^4^ in 100 μL imaging medium) were added to anti-IgG-coated coverslips and the cell-coverslip contact site was imaged by total internal reflection fluorescence (TIRF) microscopy. Images were acquired every 1 s for 10 min using an Olympus IX81 inverted microscope equipped with a 150X NA 1.45 TIRF objective, a high performance electron multiplier charge-coupled device camera (Photometrics Evolve), and real-time data acquisition software (Metamorph). Fiji software was used to quantify cell area and generate kymographs.

Stimulated emission depletion (STED) microscopy was performed as described previously (Wang et al., 2018). A20 B cells (5 × 10^4^ in 100 μL imaging medium) were allowed to spread on anti-IgG-coated coverslips before being fixed and permeabilized, as above, and then stained with Alexa Fluor 532-conjugated-phalloidin (Thermo Fisher, #A22282). STED images were acquired using a Leica TCS SP8 laser scanning STED system with a 592 nm depletion laser, a CX PL APO 100X NA 1.40 oil objective, and a Leica HyD high sensitivity detector. Huygens software (Scientific Volume Imaging, Hilversum, Netherlands) was used for image deconvolution.

### APC-Induced cSMAC Formation and BCR Signaling

B cell-APC interactions were analyzed as described previously (Wang et al., 2018; Bolger-Munro et al., 2019). Ag-bearing APCs were generated by transiently transfecting COS-7 cells (ATCC, #CRL-1651) with a plasmid encoding the mHEL-HaloTag Ag. The mHEL-HaloTag protein contains the complete HEL protein in its extracellular domain, the transmembrane and cytosolic domains of the H-2K^b^ protein, and the HaloTag protein fused to the C-terminus of the H-2K^b^ cytosolic domain (Wang et al., 2018). mHEL-HaloTag-transfected COS-7 cells (2.2 × 10^4^ cells per coverslip) were plated on glass coverslips (Thermo Fisher #12-545-100) that had been coated with 5 μg/mL fibronectin (Sigma-Aldrich, #F4759). After culturing the cells overnight, the coverslips were washed with PBS and the mHEL-HaloTag protein was labeled with the Janelia Fluor 549 HaloTag ligand (Promega, #GA1110, 1:20,000 dilution in 0.2 mL imaging medium) for 15 min at 37°C. After washing the coverslips, siRNA-transfected or inhibitor-treated B cells (5 × 10^5^ in 100 μL imaging medium) were added to the COS-7 APCs for 3–30 min at 37°C. The cells were then fixed with 4% PFA for 10 min, permeabilized with 0.1% Triton X-100 in PBS for 3 min, and blocked with 2% BSA in PBS for 30 min, all at room temperature. The cells were stained for 1 h at room temperature with an antibody that recognizes pCD79 (Cell Signaling Technologies, #5173, 1:200 in PBS + 2% FCS), washed, and then incubated for 30 min at room temperature with Alexa Fluor 647-conjugated goat anti-rabbit IgG secondary antibody (Thermo Fisher, #A21244, 1:400 in PBS + 2% FCS) plus Alexa Fluor 488-conjugated phalloidin (Thermo Fisher, #A12379, 1:400). Coverslips were mounted onto slides and the B cell-APC interface was imaged by spinning disk confocal microscopy. For each B cell, custom Fiji macros^1^ were used to quantify the total amount of pCD79 fluorescence and mHEL-HaloTag fluorescence present in clusters at the B cell-APC interface, as well as the Ag fluorescence intensity for each microcluster on an individual B cell. A cell was deemed to have formed a cSMAC when >90% of the clustered Ag fluorescence had been gathered into one or two large clusters at the center of the synapse, as defined previously (Bolger-Munro et al., 2019).

### Statistical Analysis

Two-tailed paired *t*-tests were used to compare mean values for matched sets of samples. The Mann-Whitney *U* test was used to compare ranked values in samples with many cells and high variability (e.g., dot plots for immunofluorescence signaling data). Robust Regression and Outlier Removal (ROUT) was implemented in GraphPad Prism, with Q set to 1%, in order to remove outliers (Motulsky and Brown, 2006).

## Results

### Targeting Cofilin and Its Regulators

**Figure 1:**
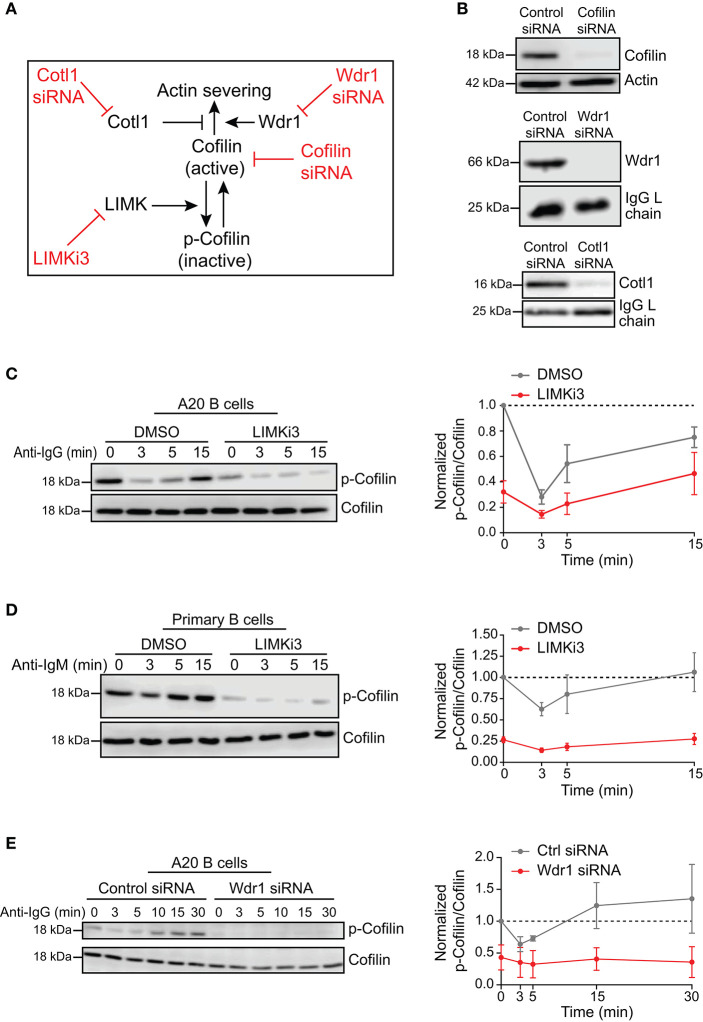
Loss-of-function approaches for modulating cofilin activation and cofilin-mediated actin severing. **(A)** To complement siRNA-mediated depletion of cofilin, cofilin activation was enhanced by inhibiting LIMK, which phosphorylates cofilin on S3. The ability of cofilin to sever actin filaments was modulated by depleting either Wdr1 or Cotl1. **(B)** A20 B cells were transfected with control non-targeting siRNA, cofilin siRNA, Wdr1 siRNA, or Cotl1 siRNA. Cell extracts were analyzed by immunoblotting. Loading controls were actin or the endogenous Ig light (L) chain of the A20 B cells. Representative blots are shown. **(C–E)** In C, A20 B cells were pre-treated with DMSO or 50 μM LIMKi3 for 1 h before being stimulated with 20 μg/mL anti-IgG for the indicated times. In **(D)**, primary murine B cells were pre-treated with DMSO or 1 μM LIMKi3 for 1 h before being stimulated with 20 μg/mL anti-IgM. In **(E)**, A20 B cells that had been transfected with control siRNA or Wdr1 siRNA were stimulated with 20 μg/mL anti-IgG. Representative p-cofilin and total cofilin immunoblots are shown (left panels). The p-cofilin/total cofilin ratios were normalized to the ratio in unstimulated (0 min) control cells (=1.0). The means ± SEM from three independent experiments are graphed for each time point (right panels).

To investigate the role of the Wdr1-LIMK-cofilin axis in BCR-induced actin reorganization, we used four different approaches to modulate either the actin-binding capability of cofilin or the ability of cofilin to promote filament severing (Figure 1A). We used siRNA to deplete either cofilin-1, the non-muscle isoform of cofilin, or its positive co-factor Wdr1. Cotl1, which may limit cofilin-mediated severing, was also depleted using siRNA. Immunoblotting showed that transfecting A20 B-lymphoma cells with these siRNAs routinely resulted in >90% reduction in the levels of the corresponding proteins, compared to cells transfected with a control non-targeting siRNA (Figure 1B). To increase the amount of “active” cofilin that is capable of binding to actin filaments, we used LIMKi3, a pharmacological inhibitor of LIMK, the kinase that phosphorylates cofilin on S3. Treating either A20 B cells or murine splenic B cells with LIMKi3 resulted in decreased phosphorylation of cofilin on S3 (Figures 1C,D). In control B cells, the inactive S3-phosphorylated form of cofilin (p-cofilin) was present at high levels in resting cells but decreased transiently after stimulation with soluble anti-Ig antibodies that cluster the BCR, as reported previously (Freeman et al., 2011, 2015). In LIMKi3-treated B cells, p-cofilin levels were lower than in control cells both before and after anti-Ig stimulation. Thus, inhibition of LIMK by LIMKi3 increased the amount of dephosphorylated active cofilin in the cells.

Surprisingly, depleting Wdr1 in A20 B cells also resulted in cofilin activation, as indicated by a substantial reduction in the amount of inactive S3-phosphorylated cofilin (Figure 1E). Similar observations have been reported in developing zebrafish neutrophils, where Wdr1 depletion results in constitutive activation of cofilin but an accumulation of actin filaments that is likely due to reduced actin severing by cofilin when Wdr1 is absent (Bowes et al., 2019). Indeed, we show below that depleting Wdr1 largely phenocopied the effects of depleting cofilin, presumably because Wdr1 optimizes cofilin-mediated filament severing.

Finally, consistent with cofilin having a major role in severing actin filaments in B cells, flow cytometry analysis showed that the amount of F-actin per cell in cofilin siRNA-transfected A20 B cells was 121.5 ± 4.8% of that in control siRNA-transfected cells (*N* = 3 independent experiments, *p* = 0.046), even though the cells were the same size (Supplementary Table 1). Similar results were obtained using A20 D1.3 B cells, which express a HEL-specific transgenic BCR. Targeting the cofilin regulators Wdr1 and LIMK did not have statistically significant effects on total F-actin levels (Supplementary Table 1).

### The Wdr1-LIMK-Cofilin Axis Controls B Cell Spreading on Immobilized Anti-Ig

When B cells are added to anti-Ig-coated coverslips, BCR signaling initiates remodeling of the actin cytoskeleton. Arp2/3 complex-nucleated actin polymerization at the periphery exerts outward force on the cell membrane that drives the formation of broad lamellipodia-like protrusions. At the same time, F-actin is depleted from the center of the cell-substrate contract site, resulting in a distinct peripheral ring of branched F-actin. In addition to mimicking the initial stages of B cell-APC interactions, this system provides a robust discovery platform for identifying proteins that regulate BCR-induced actin remodeling.

**Figure 2:**
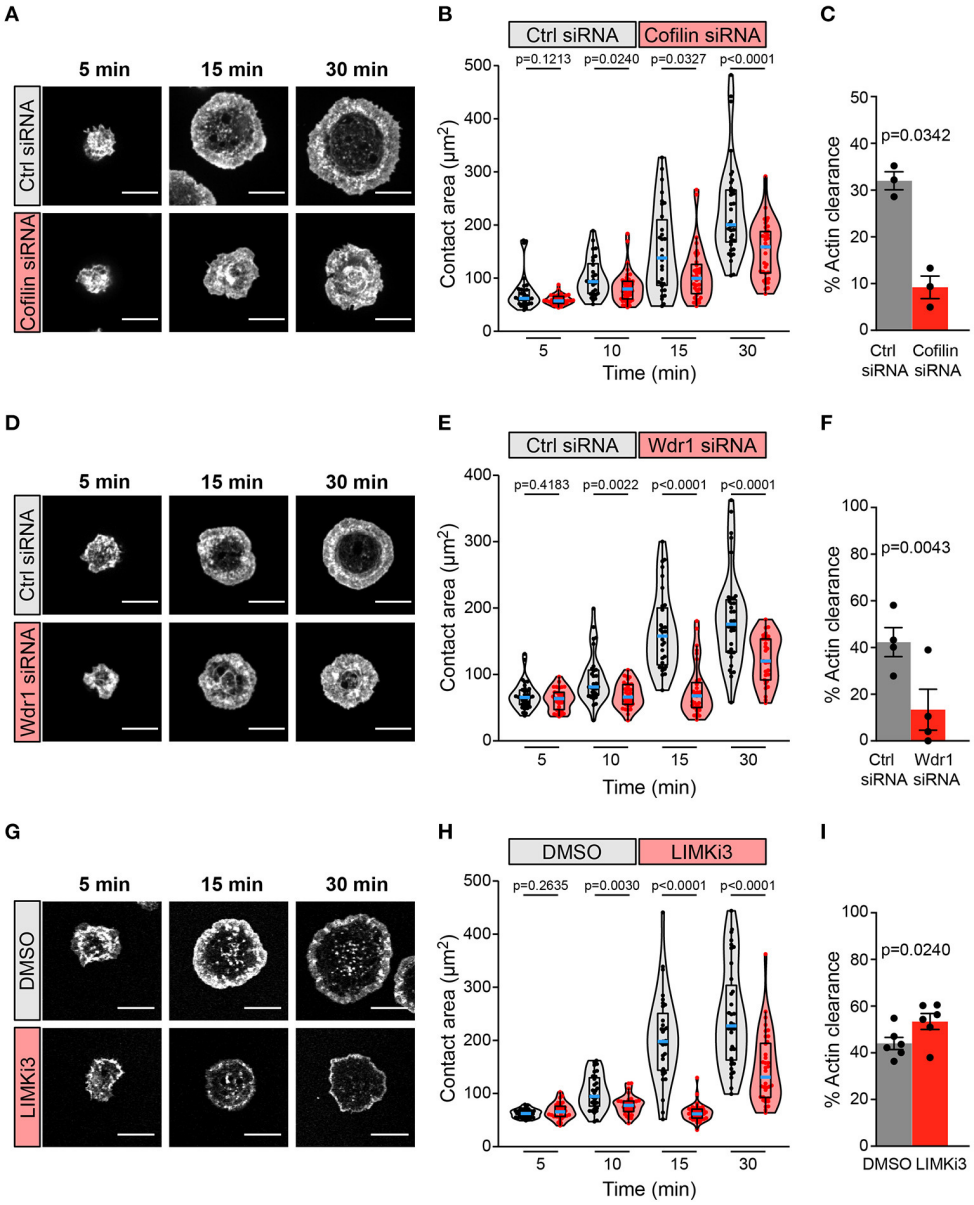
The Wdr1-cofilin-LIMK axis regulates B-cell spreading on immobilized anti-Ig. A20 B cells were transfected with control (Ctrl) siRNA or cofilin siRNA **(A–C)**, transfected with control (Ctrl) siRNA or Wdr1 siRNA **(D–F)**, or pre-treated with DMSO or 50 μM LIMKi3 for 1 h **(G–I)**. The cells were then allowed to spread on anti-IgG-coated coverslips for the indicated times before being stained with rhodamine-phalloidin and imaged by confocal microscopy. Representative images are shown **(A,D,G)**. Scale bars: 10 μm. In **(B,E,H)** the cell area was quantified using the actin staining to define the cell edge. Each dot in the beeswarm plots represents one cell and the median (blue line) and interquartile ranges (black box) for >30 cells are shown for each time point. Representative data from one of three **(B)**, four **(E)**, or six **(H)** independent experiments are shown. p-values were determined using the Mann-Whitney *U* test. In **(C,F,I)** the percent of the total cell area at the substrate contact site that was cleared of F-actin after 30 min was quantified by using the actin staining to define both the outer cell edge and the inner face of the peripheral actin ring that surrounds the central actin-depleted region of the cell (see Supplementary Figure 1 for examples). For each experiment, the median percent actin clearance was determined for each treatment group. Representative experiments are shown in Supplementary Figure 1. The bar graphs show the means ± SEM for these median values from three **(C)**, four **(F)**, or six **(I)** independent experiments, each of which is represented by a dot. p-values were determined using a two-tailed paired *t*-test.

**Figure 3:**
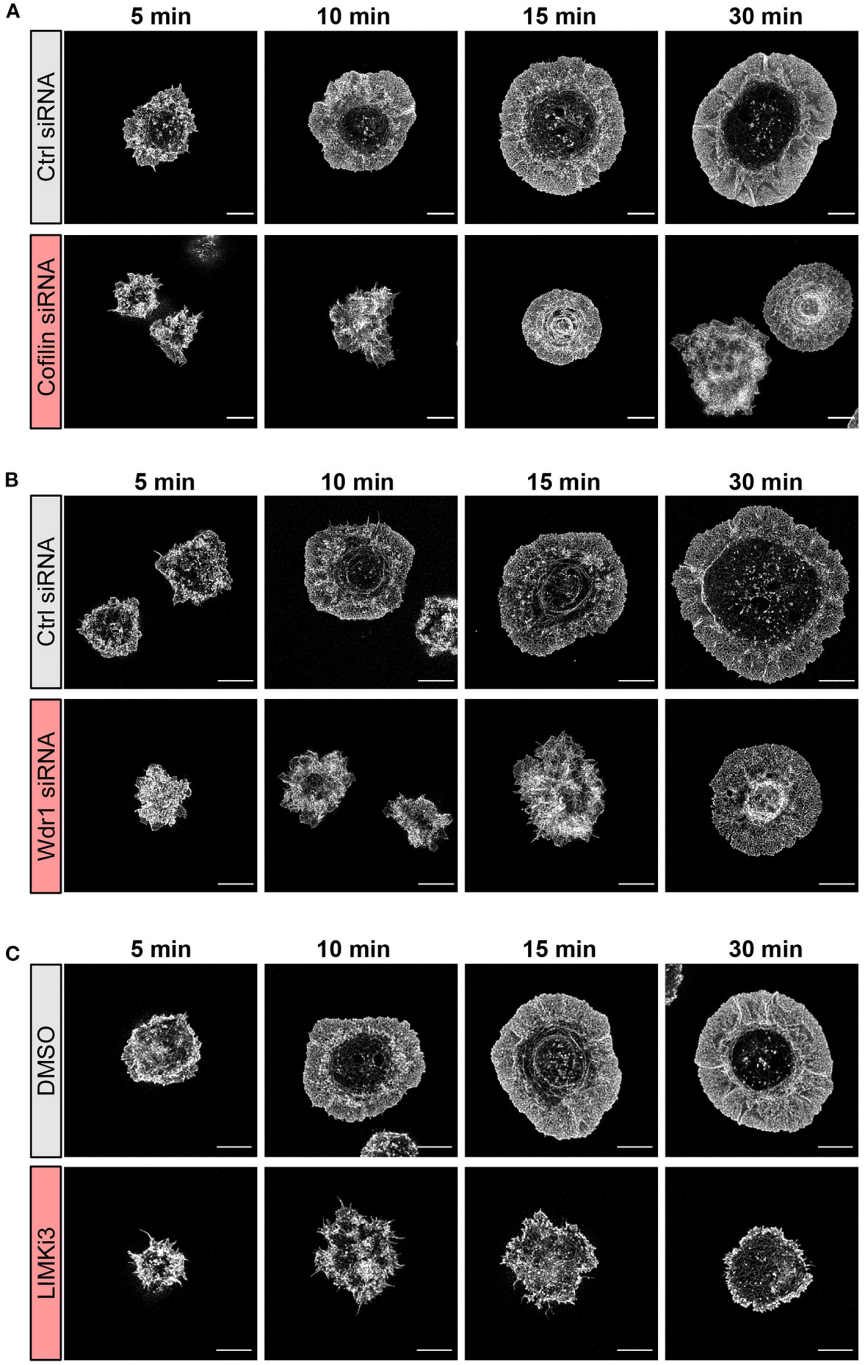
The Wdr1-LIMK-cofilin network shapes actin network architecture in B cells spreading on immobilized anti-IgG. A20 B cells were **(A)** transfected with control (Ctrl) siRNA or cofilin siRNA, **(B)** transfected with control (Ctrl) siRNA or Wdr1 siRNA, or **(C)** pre-treated with DMSO or 50 μM LIMKi3 for 1 h. The cells were then allowed to spread on anti-IgG-coated coverslips for the indicated times before being fixed, stained for F-actin, and imaged by STED microscopy. Representative images are shown. Scale bars: 5 μm.

We found that siRNA-mediated depletion of cofilin greatly impaired BCR-induced cell spreading and actin remodeling when A20 B cells were added to anti-IgG-coated coverslips (Figures 2A–C). The cofilin siRNA-transfected cells had significantly smaller substrate contact areas at the 10, 15, and 30 min time points than cells that were transfected with a control non-targeting siRNA (Figures 2A,B). BCR-induced actin reorganization was also dramatically altered. After 15 and 30 min of contact with the anti-IgG-coated coverslips, control A20 B cells developed a dense F-actin ring at the periphery of the substrate contact site while the central region of the contact site was relatively devoid of F-actin structures (Figure 2A; see also Figure 3A). Many of the cofilin siRNA-transfected A20 B cells did not effectively clear F-actin from the central region of the contact site. To quantify this, we calculated the percent of the total cell area that was depleted of actin filaments after 30 min of spreading. Single-cell analysis from one representative experiment showed that virtually all control siRNA-transfected A20 B cells cleared actin from at least 20% of the total cell-substrate contact area (median = 32%; Supplementary Figures 1A,B). In contrast, the cofilin siRNA-transfected cells from the same experiment had a bimodal distribution in which some cells cleared actin from >20% of the contact area but the majority of the cells exhibited reduced actin clearance and a substantial fraction did not clear actin from the center of the contact site (median = 9% of contact area cleared of actin; Supplementary Figures 1A,B). It is possible that the siRNA-mediated depletion of cofilin may have been incomplete in some of the cells that exhibited normal actin clearance. Nevertheless, comparing the median percent actin clearance from three experiments showed that transfection with cofilin siRNA resulted in a highly reproducible reduction in actin clearance (Figure 2C).

A20 B cells that had been transfected with Wdr1 siRNA also exhibited a significantly impaired spreading response at 10, 15, and 30 min after addition to anti-IgG-coated coverslips, and this was associated with reduced actin clearance at the center of the contact site (Figures 2D–F; see also Figure 3B). Single-cell analysis from one representative experiment showed that almost all control siRNA-transfected A20 B cells cleared actin from more than 20% of the total contact area (median = 43%; Supplementary Figures 1C,D) whereas Wdr1 siRNA-transfected cells exhibited a bimodal distribution with a substantially lower median percent actin clearance (median = 10%; Supplementary Figures 1C,D), similar to cofilin siRNA-transfected cells. Nevertheless, the median percent actin clearance was consistently and significantly reduced in four experiments comparing control siRNA- and Wdr1 siRNA-transfected A20 B cells (Figure 2F). Because siRNA transfection does not result in complete protein depletion in all cells, we could be underestimating the effect of complete loss of Wdr1. Thus, depleting either cofilin or Wdr1 results in decreased cell spreading, reduced actin clearance, and increased thickness of the peripheral actin ring. These findings are consistent with the idea that cofilin-mediated actin severing is essential for the BCR-induced actin reorganization that drives cell spreading and that Wdr1 is required for the cofilin-mediated severing of actin filaments.

Based on these findings we hypothesized that enhancing cofilin activity by inhibiting its negative regulator, LIMK, would increase B cell spreading. Surprisingly, pre-treating A20 B cells with LIMKi3 significantly reduced B cell spreading on anti-IgG-coated coverslips (Figures 2G,H). However, in striking contrast to depleting either cofilin or Wdr1 depletion, LIMKi3-treated B cells exhibited a thinner peripheral actin ring (Figure 2G; see also Figure 3C), which corresponded with a greater percent of the cell area being cleared of actin (Figures 2G,I and Supplementary Figures 1E,F). This could be a consequence of increased cofilin-mediated actin severing at the inner face of the peripheral actin ring. These results indicate that LIMK activity regulates BCR-induced actin remodeling and that cofilin activity must be precisely regulated in order to maximize the branched actin polymerization that drives B cell spreading.

Because Cotl1 competes with cofilin for binding to F-actin, we hypothesized that depleting Cotl1 would result in increased cofilin-mediated actin severing and reproduce the effect of inhibiting LIMK. However, we found that substantial depletion of Cotl1 (Figure 1B) had no effect on the ability of A20 B cells to spread on immobilized anti-IgG (Supplementary Figure 2). Thus, Cotl1 is not an essential, non-redundant regulator of BCR-induced actin remodeling, at least in A20 B cells.

To gain further insights into how targeting the Wdr1-LIMK-cofilin axis impairs BCR-induced cell spreading we used STED super-resolution microscopy to visualize changes in actin network architecture. When control A20 B cells were added to anti-IgG-coated coverslips, protrusions containing branched actin networks was first observed at 5 min, concomitant with the appearance of a central actin-depleted region (Figures 3A–C). By 10 min, the cells had assembled a thick peripheral ring of branched actin. As well, linear actin filaments formed into arc-like structures that were parallel to the inner face of peripheral actin ring and surrounded the actin-depleted region at the center of the contact site. Actin arcs, which are often associated with myosin, have been observed at T- and B-cell immune synapses (Murugesan et al., 2016; Bolger-Munro et al., 2019). When cofilin was depleted, the A20 B cells were unable to clear actin from the center of the contact site. Instead, many of these cells accumulated concentric actin arc-like structures and rings at the center of the contact site (Figure 3A). Wdr1-depleted A20 B cells also exhibited defective actin clearance at the center of the contact site, although some of these cells did not organize actin into concentric arc-like structures at the center of the contact site as extensively as the cofilin-depleted A20 B cells (Figure 3B). Conversely, A20 B cells treated with the LIMK inhibitor exhibited larger actin-depleted central regions and much thinner peripheral rings of branched actin than control cells (Figure 3C). Interestingly, depleting cofilin or Wdr1, as well as increasing cofilin activity by inhibiting LIMK, delayed the ability of A20 B cells to initiate actin reorganization and form a peripheral ring of branched actin (Figures 3A–C). Control cells formed a distinct peripheral actin ring surrounding an actin-depleted central region within 5–10 min of being added to anti-IgG-coated coverslips. In contrast, when the Wdr1-LIMK-cofilin axis was perturbed, the peripheral actin structures were disorganized at the earlier time points and a distinct peripheral ring of branched actin did not develop until the 15 or 30 min time points. This altered peripheral actin architecture was associated with decreased cell spreading. Thus, properly regulated cofilin activity is important for establishing the peripheral branched actin structures that drive B cell spreading on rigid substrates.

Finally, we ruled out the possibility that the impaired anti-IgG-induced spreading caused by targeting Wdr1, cofilin, or LIMK was due to reductions in BCR cell surface levels or BCR signaling, as opposed to specific effects on actin remodeling. Flow cytometry showed that targeting Wdr1, cofilin, or LIMK did not have a significant effect on the amount of IgG-BCRs on the surface of A20 B cells and did not alter the size of the cells (Supplementary Table 1). We also analyzed BCR signaling in response to soluble anti-Ig antibodies, which is much less dependent on actin dynamics and organization than BCR signaling in response to APC-bound Ags that induce immune synapse formation (Bolger-Munro et al., 2019). An essential initial event in BCR signaling is phosphorylation of the immunoreceptor tyrosine-based activation motifs (ITAMs) in the CD79a/CD79b subunit of the BCR by the Lyn and Syk tyrosine kinases (Packard and Cambier, 2013). This enables the formation of signaling complexes that lead to downstream signaling reactions including ERK activation and increases in cytoplasmic Ca^2+^. In A20 B cells, we found that depleting cofilin, depleting Wdr1, or treating the cells with LIMKi3 had no effect on the ability of soluble anti-Ig antibodies to stimulate the phosphorylation of CD79a/CD79b or ERK (Supplementary Figure 3). Similarly, depleting either cofilin or Wdr1 in A20 B cells or in the HEL-specific A20 D1.3 B cells did not alter Ca^2+^ responses to soluble anti-Ig antibodies (Supplementary Figure 4).

### The Wdr1-LIMK-Cofilin Axis Regulates Actin Dynamics in B Cells

**Figure 4:**
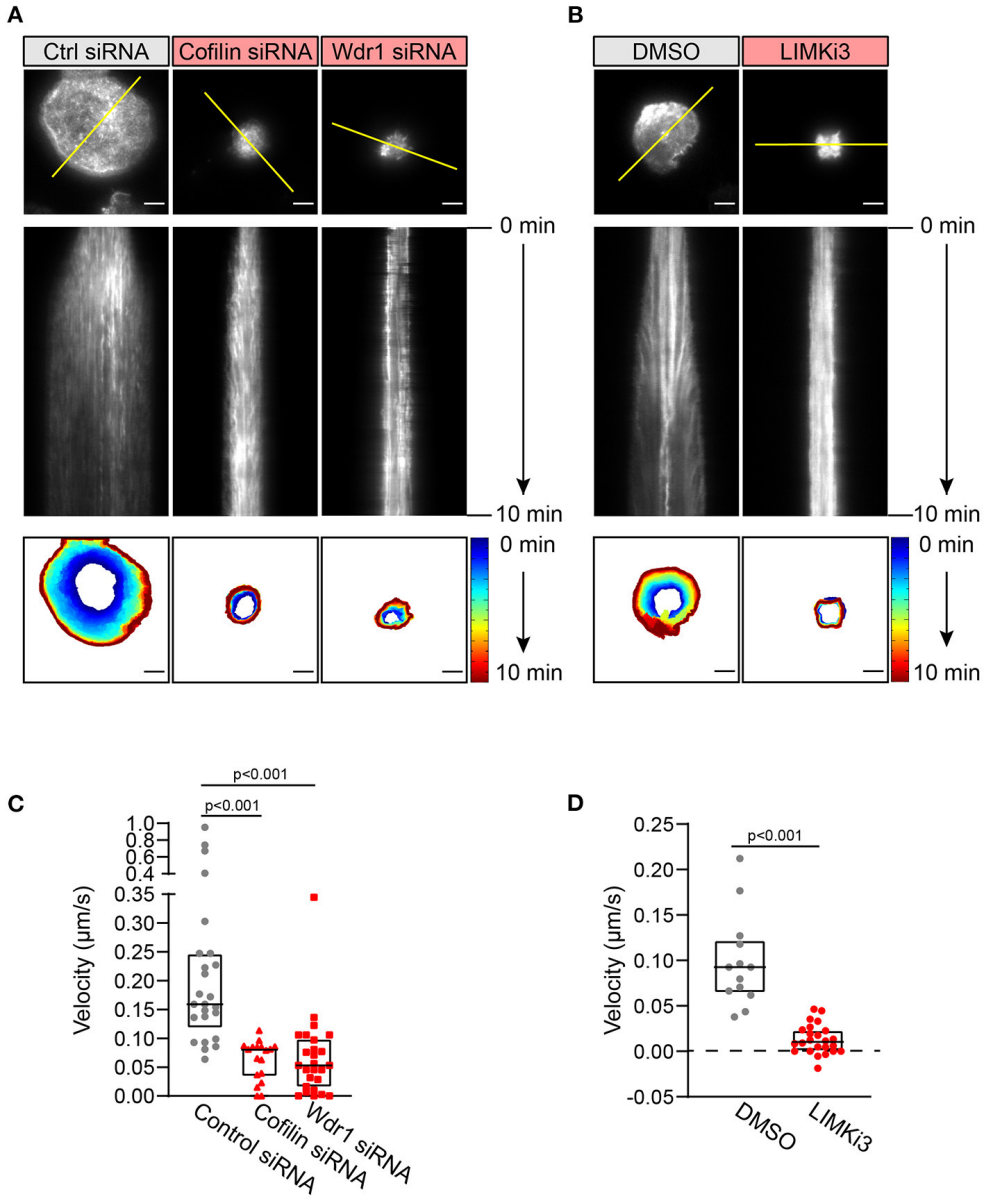
The Wdr1-LIMK-cofilin network is important for BCR-induced actin dynamics. A20 B cells were co-transfected with F-tractin-GFP cDNA and either control (Ctrl) siRNA, cofilin siRNA, or Wdr1 siRNA **(A)**, or transfected with F-tractin-GFP cDNA and then pre-treated with DMSO or 50 μM LIMKi3 for 1 h **(B)**. The cells were then added to anti-IgG-coated coverslips and imaged by TIRF microscopy at 1 s intervals for 10 min. The top panels are the final frames, i.e., 10 min time point, from Movie 1 (control siRNA), Movie 2 (cofilin siRNA), Movie 3 (Wdr1 siRNA), Movie 4 (DMSO-treated), and Movie 5 (LIMKi3-treated). The middle panels are kymographs along the yellow lines in the top panels. Static F-actin structures appear as vertical lines. In the bottom panels, the cell edge in each frame, as defined by F-actin staining, was overlaid as a temporally-coded time series. Scale bars: 5 μm. **(C,D)** The centripetal velocity (Δx/Δt) was calculated for individual actin tracks on kymographs. Each dot on the graphs is an individual actin track. For each condition the velocity was determined for 13-26 actin tracks from 5 to 10 cells. The median and interquartile ranges are shown. The Mann-Whitney *U* test was used to calculate p-values.

To visualize how cofilin, Wdr1, and LIMK modulate BCR-induced actin remodeling in real time, A20 B cells were transfected with F-tractin-GFP, a fluorescent fusion protein that binds dynamically to F-actin. The cells were either co-transfected with cofilin or Wdr1 siRNAs, or treated with LIMKi3, prior to being added to anti-IgG-coated coverslips and imaged by TIRF microscopy. We found that cofilin depletion, Wdr1 depletion, and LIMK inhibition all resulted in reduced spreading, as shown in Figure 2, and that this was accompanied by impaired peripheral actin dynamics (Figure 4). Arp2/3 complex-nucleated branch actin polymerization at the plasma membrane exerts outward forces that are opposed by the elastic resistance of the plasma membrane. This results in actin retrograde flow toward the center of the cell, which can be visualized by kymograph analysis. This actin retrograde flow was evident in control A20 B cells spreading on anti-IgG-coated coverslips (Figures 4A,B and Supplementary Movies 1, 4), as we have shown previously (Bolger-Munro et al., 2019). However, when cofilin or Wdr1 were depleted (Figure 4A and Supplementary Movies 2, 3), and when LIMK was inhibited (Figure 4B and Supplementary Movie 5), the peripheral actin network was relatively static and the retrograde actin flow was substantially reduced compared to control cells. To quantify this, we used the kymographs to calculate the centripetal velocity (Δx/Δt) for multiple actin tracks. This analysis showed that targeting cofilin, Wdr1, or LIMK reduced the median velocity of the actin retrograde flow by 50, 67, and 89%, respectively (Figures 4C,D). Thus, interfering with the Wdr1-LIMK-cofilin regulatory network inhibits the peripheral actin dynamics that occur when B cells spread on anti-Ig-coated coverslips.

### APC-Induced cSMAC Formation and CD79 Phosphorylation Is Regulated by the Wdr1-LIMK-Cofilin Axis

When B cells interact with Ag-bearing APCs, Arp2/3 complex-dependent actin dynamics drives the centripetal movement and coalescence of BCR-Ag microclusters, which amplifies microcluster-based BCR signaling and leads to cSMAC formation (Bolger-Munro et al., 2019). This Arp2/3 complex-dependent amplification of microcluster-associated CD79 phosphorylation is most evident during the first 10 min after adding B cells to APCs. Importantly, a combination of wash-in and wash-out experiments with the Arp2/3 complex inhibitor CK-666 showed that ablating this BCR signal amplification during the initial stages of B cell-APC interaction impaired subsequent B cell activation responses. Because cofilin-mediated actin disassembly may support Arp2/3 complex-nucleated actin polymerization by recycling actin monomers and Arp2/3 complexes, we asked whether the Wdr1-LIMK-cofilin axis regulates B cell responses to APC-bound Ags.

To study Ag-specific B cell-APC interactions, A20 D1.3 B cells with a HEL-specific BCR were added to COS-7 APCs expressing the fluorescently-labeled mHEL-HaloTag Ag on their surface. The mHEL-HaloTag Ag is a transmembrane protein that contains the complete HEL protein in its extracellular domain. After 3–30 min of B cell-APC interaction, the cells were fixed and stained with an antibody that recognizes the phosphorylated CD79a/CD79b ITAMs in the cytoplasmic domains of the BCR signaling subunit. Ag-induced phosphorylation of the CD79a/CD79b ITAMs is an essential early event in BCR signaling. Imaging the B cell-APC interface allowed us to visualize BCR-Ag microclusters, monitor their coalescence into a cSMAC, and quantify the amount of pCD79 and mHEL-HaloTag Ag fluorescence that was present in microclusters.

**Figure 5:**
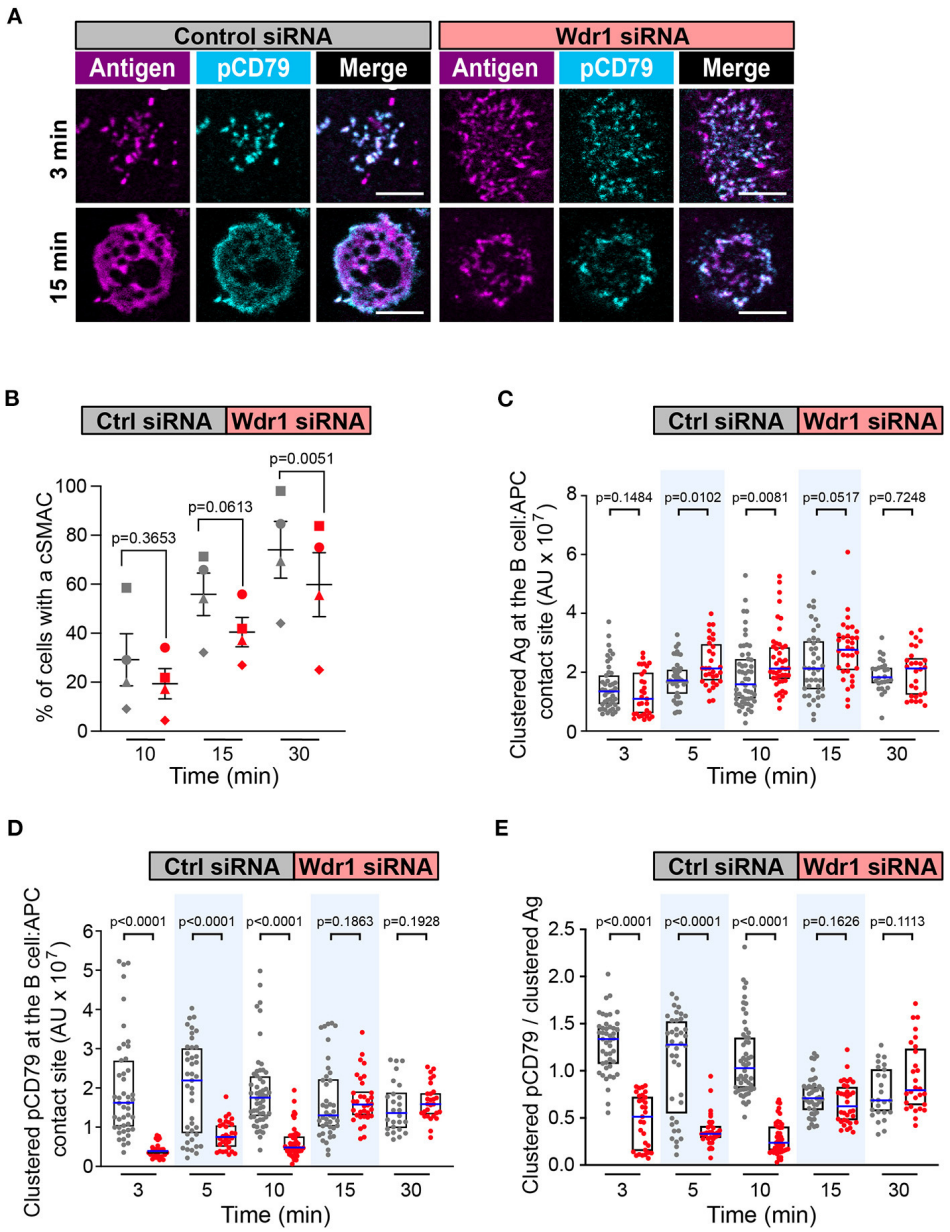
Wdr1 is important for cSMAC formation and BCR signaling at the immune synapse. A20 D1.3 B cells that had been transfected with control (Ctrl) siRNA or Wdr1 siRNA were added to mHEL-HaloTag-expressing COS-7 APCs. The cells were then fixed at the indicated times and the B cell-APC interface was imaged by spinning disk microscopy. **(A)** Representative images. Scale bars: 5 μm. **(B)** For each time point, the percent of cells that had formed a cSMAC, defined as >90% of the total Ag fluorescence intensity being contained in 1-2 clusters, is graphed. Each symbol on the graph represents an independent experiment. Paired *t*-tests were used to calculate p-values. **(C,D)** The total fluorescence intensity of the mHEL-HaloTag Ag **(C)** or pCD79 **(D)** that was present in clusters at the B cell-APC contact site was quantified for each cell. Each dot is one cell. n >25 cells per condition. The median (blue line) and interquartile ranges (black box) are shown. **(E)** For each B cell represented in **(C,D)**, the total fluorescence intensity of clustered pCD79 was divided by the total fluorescence intensity of clustered Ag. The ratio is graphed. The median (blue line) and interquartile ranges (black box) are shown. The data in **(C–E)** are from the same experiment, which is representative of four independent experiments. The Mann-Whitney *U* test was used to calculate p-values for **(C–E)**.

We focused first on Wdr1 because its role in B-cell immune synapse formation and APC-induced BCR signaling has not been investigated. When control siRNA-expressing A20 D1.3 B cells were added to mHEL-HaloTag-expressing APCs, BCR-Ag microclusters formed rapidly at the B cell-APC contact site and co-localized with pCD79 clusters (Figure 5A). By 30 min, nearly all of the cells had coalesced BCR-Ag microclusters into a cSMAC (Figure 5B), which we define as >90% of the Ag fluorescence being contained in 1-2 large clusters at the center of the synapse. For this analysis, we quantified the Ag fluorescence intensity that was associated with each discrete cluster on an individual B cell and then expressed it as a percent of the total Ag fluorescence intensity for all clusters on that cell. The distribution of total Ag fluorescence intensity into individual clusters is shown for multiple cells in Supplementary Figure 5. When Wdr1 was depleted using siRNA, BCR-Ag microclusters formed at the B cell-APC contact site (Figure 5A). However, the percent of cells that formed a cSMAC was consistently lower than in control cells (Figure 5B and Supplementary Figure 5A), even though the Wdr1-depleted cells gathered more Ag into clusters at some time points (Figure 5C). In Figure 5A, the Wdr1 siRNA-transfected cell shown for the 15 min time point exemplifies a cell that did not form a cSMAC. The Ag fluorescence associated with this cell is distributed among multiple discrete clusters.

Importantly, the initial microcluster-based BCR signaling at the immune synapse was significantly reduced when Wdr1 was depleted. The amount of clustered pCD79 at the contact site with the APC was significantly lower in the Wdr1 siRNA-transfected A20 D1.3 B cells than in the control siRNA-transfected cells at the 3, 5, and 10 min time points (Figure 5D). Because Wdr1 depletion did not decrease the total amount of Ag that was gathered into clusters (Figure 5C), the decreased levels of pCD79 at the B cell-APC interface were not due to impaired formation of BCR-Ag microclusters. Indeed, calculating the ratio of clustered pCD79 divided by clustered Ag for each B cell revealed that the BCR signaling output per unit of Ag that was gathered into clusters (i.e., signal amplification) was significantly reduced at the 3, 5, and 10 min time points when Wdr1 was depleted (Figure 5E). Thus, for membrane-bound Ags, Wdr1 contributes to mechanisms that amplify CD79 phosphorylation. This is in contrast to B cell responses to soluble BCR ligands, where depleting Wdr1 had no effect on anti-Ig-induced CD79 phosphorylation, Erk phosphorylation, or Ca^2+^ flux (Supplementary Figures 3, 4).

**Figure 6:**
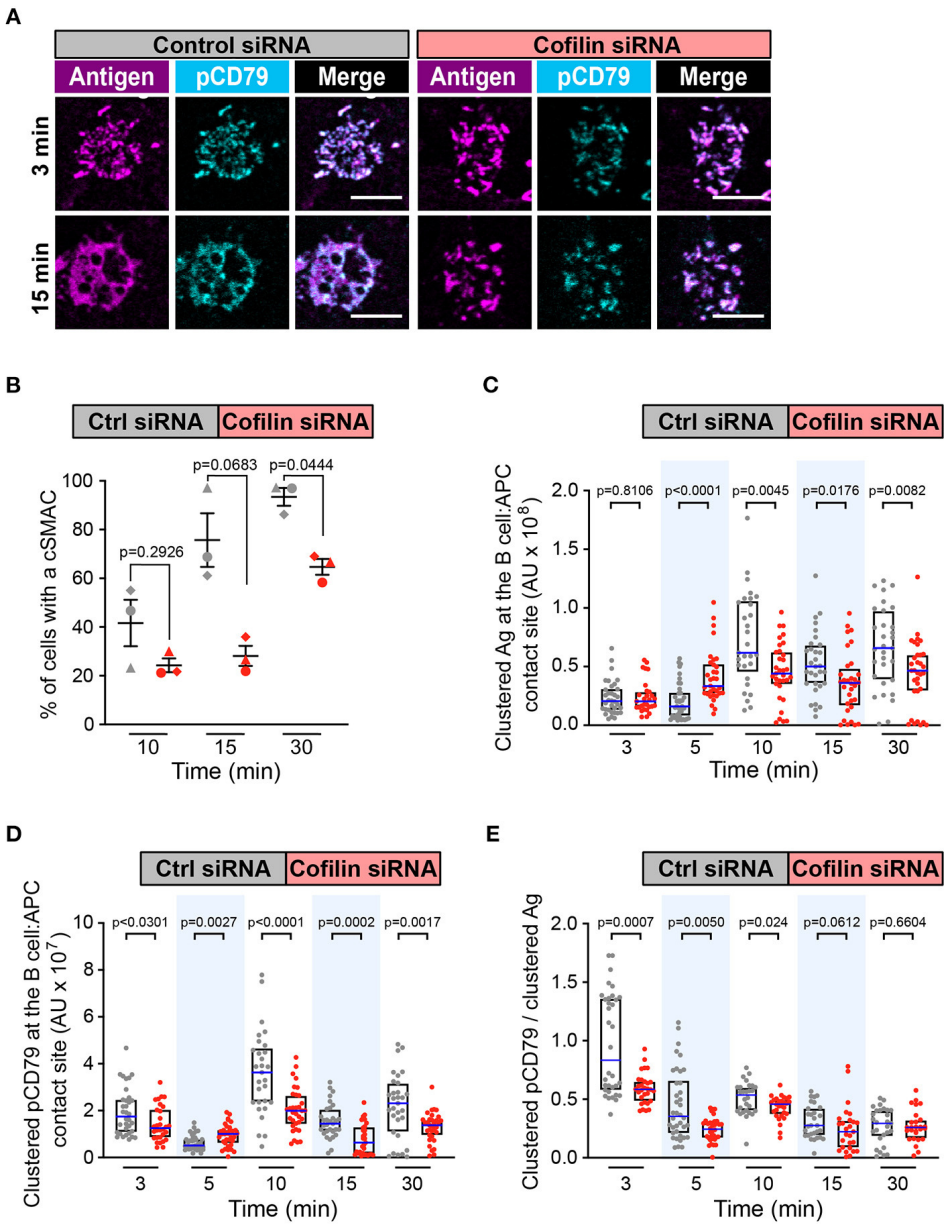
Cofilin is important for cSMAC formation and BCR signaling at the immune synapse. A20 D1.3 B cells that had been transfected with control (Ctrl) siRNA or cofilin siRNA were added to mHEL-HaloTag-expressing COS-7 APCs. The cells were then fixed at the indicated times and the B cell-APC interface was imaged by spinning disk microscopy. **(A)** Representative images. Scale bars: 5 μm. **(B)** For each time point, the percent of cells that had formed a cSMAC is graphed. Each symbol represents an independent experiment. Paired *t*-tests were used to calculate p-values. **(C,D)** The total fluorescence intensity of the mHEL-HaloTag Ag **(C)** or pCD79 **(D)** that was present in clusters at the B cell-APC contact site was quantified for each cell. Each dot is one cell. n >30 cells per condition. The median (blue line) and interquartile ranges (black box) are shown. **(E)** For each B cell represented in **(C,D)**, the total fluorescence intensity of clustered pCD79 was divided by the total fluorescence intensity of clustered Ag. The median (blue line) and interquartile ranges (black box) are shown. The data in **(C–E)** are from the same experiment, which is representative of three independent experiments. The Mann-Whitney *U* test was used to calculate p-values for **(C–E)**.

Because Wdr1 optimizes the actin-severing capabilities of cofilin, we then asked whether depleting cofilin also impaired cSMAC formation and APC-induced BCR signaling. Cofilin-depleted cells rapidly formed BCR-Ag microclusters (Figure 6A) but the percent of cells that formed a cSMAC after 30 min was reduced compared to control cells (Figure 6B and Supplementary Figure 5B), as was the case when Wdr1 was depleted. Depleting cofilin appeared to alter the kinetics of BCR-induced Ag clustering. Compared to control siRNA-transfected cells, the amount of Ag that was gathered into clusters was higher in the cofilin-depleted cells after 5 min but significantly lower at 10, 15, and 30 min (Figure 6C). Importantly, the cofilin-depleted cells had decreased amounts of clustered pCD79 at the B cell-APC interface at the 3, 10, 15, and 30 min time points (Figure 6D). The amount of clustered pCD79 at the 5 min time point was greater in the cofilin-depleted cells than in the control cells, which may reflect the increased amount of Ag that was gathered into BCR-Ag microclusters at that time point. Nevertheless, BCR signal amplification, the amount of clustered pCD79 per unit of Ag that was gathered into BCR-Ag microclusters per cell was significantly reduced at the 3, 5, and 10 min time points in the cofilin-depleted cells and trended lower at the 15 and 30 min time points (Figure 6E). Thus, at early time points (3 min and 5 min), the loss of cofilin strongly decreases BCR signaling amplification without decreasing Ag gathering. At the later time points (10, 15, and 30 min), both reduced Ag gathering and reduced BCR signal amplification appear to contribute to the decreased pCD79 levels at the B cell-APC interface in the cofilin siRNA-transfected cells. In contrast, depleting cofilin had no effect on the ability of soluble anti-Ig to stimulate CD79 phosphorylation, Erk phosphorylation, or Ca^2+^ flux (Supplementary Figures 3, 4). Thus, cofilin and Wdr1 enhance BCR signaling in response to membrane-bound Ags but are not essential for BCR signaling in response to soluble ligands that are uniformly distributed in the surrounding medium.

In T cells, Cotl1 is recruited to the immune synapse where it enhances the formation of lamellipodia that spread across the surface of the APC (Kim et al., 2014). However, we found that depleting Cotl1 did not affect APC-induced cSMAC formation or BCR signal amplification at the immune synapse (data not shown).

**Figure 7:**
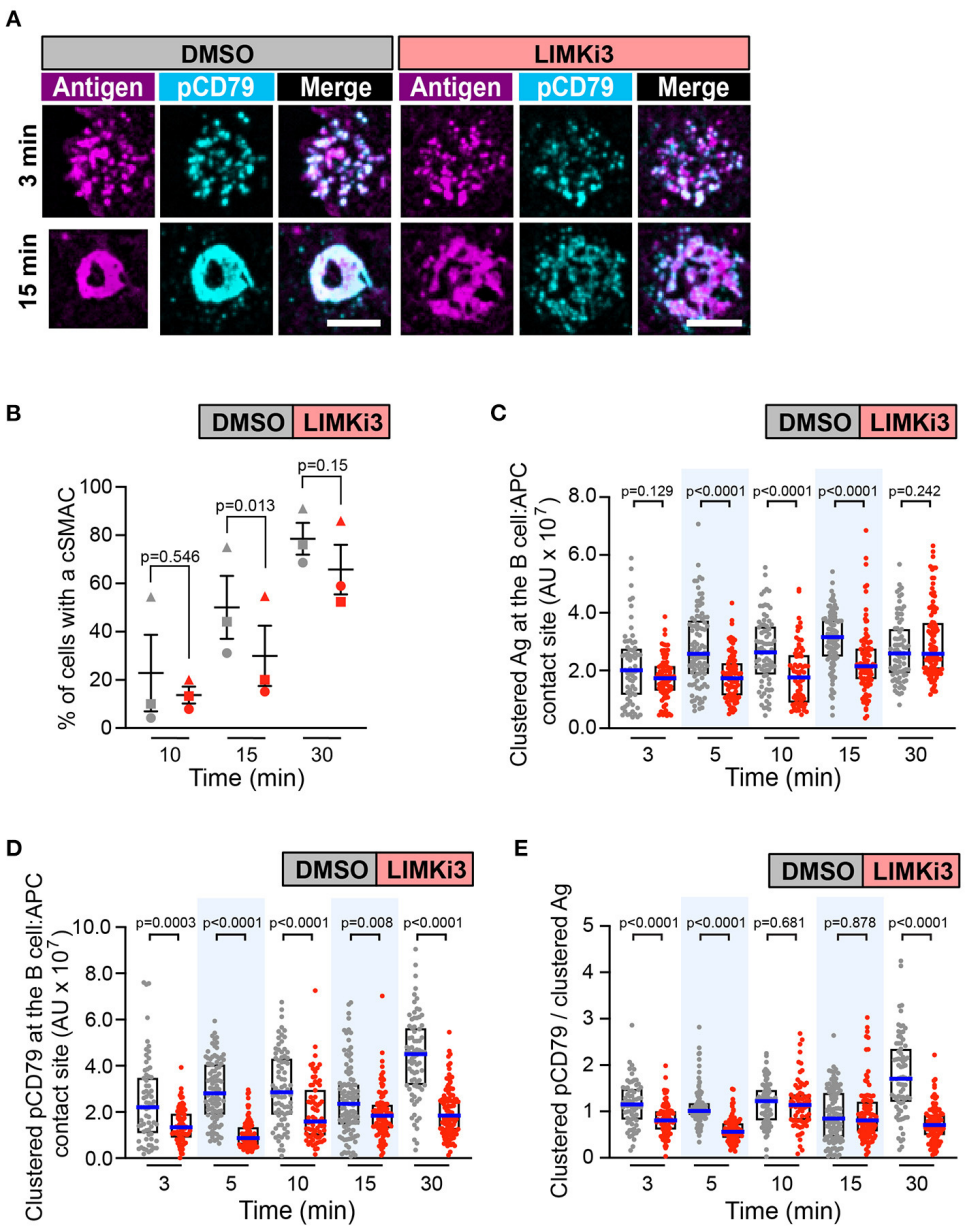
Inhibiting LIMK in A20 D1.3 B cells impairs cSMAC formation and BCR signaling at the immune synapse. A20 D1.3 B cells were pre-treated for 1 h with DMSO or 50 μM LIMKi3 before being added to mHEL-HaloTag-expressing COS-7 APCs. The cells were then fixed at the indicated times and the B cell-APC interface was imaged by spinning disk microscopy. **(A)** Representative images. Scale bars: 5 μm. **(B)** For each time point, the percent of cells that had formed a cSMAC is graphed. Each symbol represents an independent experiment. Paired *t*-tests were used to calculate p-values. **(C,D)** The total fluorescence intensity of the mHEL-HaloTag Ag **(C)** or pCD79 **(D)** that was present in clusters at the B cell-APC contact site was quantified for each cell. Each dot is one cell. n > 67 cells per condition. The median (blue line) and interquartile ranges (black box) are shown. **(E)** For each B cell represented in **(C,D)**, the total fluorescence intensity of clustered pCD79 was divided by the total fluorescence intensity of clustered Ag. The median (blue line) and interquartile ranges (black box) are shown. The data in **(C–E)** are from the same experiment, which is representative of three independent experiments. The Mann-Whitney *U* test was used to calculate p-values for **(C–E)**.

As a gain-of-function approach for enhancing the actions of cofilin, we treated B cells with LIMKi3 in order to reduce the ability of LIMK to phosphorylate and inactivate cofilin. When A20 D1.3 B cells were treated with LIMKi3, BCR-Ag microclusters formed throughout the contact site but cSMAC formation was slightly reduced compared to control cells (Figures 7A,B and Supplementary Figure 5C). Moreover, the gathering of Ag into clusters was significantly reduced at the 5, 10, and 15 min time points in the LIMKi3-treated cells (Figure 7C). Importantly, the amount of clustered pCD79 present at the B cell-APC interface was significantly lower at all time points when LIMK was inhibited (Figure 7D). This reflects a combination of reduced BCR signal amplification at the 3, 5, and 30 min time points (Figure 7E) and the reduced Ag gathering that occurs at 5, 10, and 15 min (Figure 7C). Similar trends were observed in primary B cells from MD4 mice, which express a transgenic HEL-specific BCR (Supplementary Figure 6). LIMKi3 treatment of *ex vivo* MD4 B cells resulted in reduced Ag gathering at the 10, 15, and 30 min time points, substantially reduced pCD79 levels at 3 and 30 min, and reduced BCR signaling per unit of gathered Ag at the 3, 15, and 30 min time points. Thus, LIMKi3 treatment modulates both Ag gathering and BCR signaling amplification, resulting in decreased BCR signaling at the immune synapse at several time points. As for cofilin and Wdr1, targeting LIMK did not impair BCR signaling in response to soluble anti-Ig antibodies (Supplementary Figure 3). Overall, we found that targeting cofilin, Wdr1, or LIMK all resulted in decreased BCR signaling in response to APC-bound Ags. Although the exact time course of the APC-induced CD79 phosphorylation varied from experiment-to-experiment, both total pCD79 levels and the signal amplification parameter (clustered pCD79/clustered Ag) were routinely decreased at multiple time points over the first 30 min of B cell-APC encounter. We have previously shown that the magnitude of BCR signaling during the first 30 min of B cell-APC interaction determines whether the B cell activation program is initiated (Bolger-Munro et al., 2019).

## Discussion

Cellular processes that depend on actin remodeling require cofilin to disassemble existing actin networks and to support new actin polymerization. Actin filament severing by cofilin generates new barbed ends at which filament elongation can occur. Moreover, the disassembly of severed filament segments releases actin monomers, Arp2/3 complexes, and other actin-binding proteins, which can then be used for new actin assembly. The ability of cofilin to bind and sever actin filaments is controlled by a network of proteins that includes Wdr1, LIMK, and Cotl1. This regulatory network can presumably integrate input from multiple signaling pathways, enabling dynamic spatiotemporal regulation of cofilin activity. We have shown that cofilin, Wdr1, and LIMK are all essential for BCR-induced actin remodeling and cell spreading, as well as immune synapse formation and APC-induced BCR signaling. Because actin dynamics are strongly influenced by mechanical forces, it is important to point out that we analyzed actin-dependent B cell processes on both a rigid substrate coated with immobilized anti-Ig antibodies and on APCs expressing Ags that are mobile within the plasma membrane. However, B cells extend lamellipodial protrusions on both these surfaces (Bolger-Munro et al., 2019), and a common feature of lamellipodia is actin treadmilling and retrograde flow that is dependent on the concerted actions of the Arp2/3 complex and cofilin.

### The Wdr1-LIMK-Cofilin Axis Regulates BCR-Induced Actin Remodeling and Spreading

The radial spreading of B cells on immobilized anti-Ig antibodies is driven by Arp2/3 complex-nucleated actin polymerization and the resulting assembly of dendritic actin networks at the cell periphery (Bolger-Munro et al., 2019). We had previously shown that the ability of B cells to spread on immobilized anti-Ig is severely impaired when cofilin is depleted in the A20 B cell line (Wang et al., 2017). Using STED super-resolution microscopy, we have extended these findings to show that cofilin-depleted B cells form a much thicker peripheral ring of branched actin than control cells and are unable to clear actin from the center of the contact site. A similar disruption of actin organization is observed when cofilin is depleted in developing neurons (Flynn et al., 2012). Developing neurons adopt a similar morphology as a spreading B cell, with a dense actin network at the periphery and an actin-depleted region at the center of the substrate contact site. Cofilin deficiency in these cells leads to “congestion of the intracellular space” as well as impaired neurite outgrowth. Similarly, localized inactivation of cofilin in the lamellipodia of neuronal cells results in expansion of the peripheral actin ring, which results from decreased filament disassembly (Vitriol et al., 2013). Thus, cofilin-mediated actin disassembly appears to be an essential prerequisite for the actin remodeling that drives membrane protrusion and cell spreading. Consistent with this idea, using jasplakinolide to stabilize actin filaments prevents B cell spreading (Freeman et al., 2011).

Wdr1 optimizes the spacing of cofilin molecules on actin filaments such that filament severing is favored (Elam et al., 2013; Gressin et al., 2015; Tanaka et al., 2018). Hence, depleting Wdr1 reduces cofilin-mediated actin severing and often phenocopies cofilin depletion. In *Drosophila*, depleting either the Wdr1 homolog *flare* or the cofilin homolog *twinstar* results in similar phenotypes (Ren et al., 2007; Chu et al., 2012). Both *flare* mutants and *twinstar* mutants exhibit an accumulation of actin filaments and an increase in actin network stability. Consistent with this, we found that Wdr1-depleted B cells exhibited a similarly impaired spreading phenotype as cofilin-depleted B cells, with an expanded peripheral ring of branched actin, reduced actin clearance at the center of the substrate contact site, and impaired peripheral actin dynamics. Wdr1 depletion in B cells also resulted in an increase in the amount of non-phosphorylated (activated) cofilin that is capable of binding actin filaments. In the absence of Wdr1, the saturation of actin filaments with cofilin would stabilize the filaments and prevent actin network remodeling. Indeed, depleting Wdr1 in the neutrophils of developing zebrafish, which also causes cofilin dephosphorylation, results in an accumulation of actin filaments (Bowes et al., 2019). A reduction in cofilin-mediated actin network disassembly may also limit the recycling of Arp2/3 complexes and actin monomers that supports new actin polymerization. In yeast, disrupting the gene encoding the Wdr1 homolog Aip1 reduces the concentration of actin monomers in cells (Okreglak and Drubin, 2010). Moreover, biochemical studies revealed a role for Wdr1/Aip1 in converting cofilin-generated actin oligomers into monomers (Okreglak and Drubin, 2010). Thus, Wdr1 may enhance the actions of cofilin in multiple ways to support actin treadmilling.

In contrast to the impaired spreading of Wdr1-depleted A20 B cells that we observed, peripheral blood B cells from patients with mutations that ablate Wdr1 expression exhibit enhanced spreading on immobilized anti-Ig (Pfajfer et al., 2018). However, this experiment was done in conjunction with CpG DNA stimulation of the B cells. CpG DNA is a ligand for Toll-like receptor (TLR) 9 and we have previously shown that TLR9 signaling increases actin turnover dynamics in B cells and impacts the activity of multiple actin-regulatory proteins, including cofilin (Freeman et al., 2015). Whether TLR-induced “dynamization” of the actin cytoskeleton requires Wdr1 is not known.

We found that B cell spreading was also impaired when we used the LIMK inhibitor to increase the amount of activated cofilin. In contrast to cofilin depletion and Wdr1 depletion, the reduced spreading in LIMKi3-treated cells was associated with a thinner peripheral actin ring. This may reflect an increased rate of cofilin- and Wdr1-dependent actin filament severing at the inner face of the actin ring, which exceeds the rate of actin polymerization at the plasma membrane. A peripheral branched actin network with reduced thickness may generate less outward force to drive membrane protrusion. Alternatively, dynamic spatiotemporal regulation of cofilin activity by LIMK may be essential for sustaining membrane protrusion. In Jurkat T cells, depleting either LIMK or cofilin inhibits SDF-1-induced chemotaxis, suggesting that rapidly turning cofilin on and off is important for directional cell movement (Nishita et al., 2004). LIMK-deficient Jurkat cells exhibit “immature protrusion events,” suggesting that transient suppression of cofilin activity by LIMK enables sustained assembly of actin-based membrane protrusions. This may also be critical for B cell spreading.

B cell spreading may require complex spatial and temporal control of LIMK-mediated inhibition of cofilin. Upon contacting anti-Ig- or Ag-coated surfaces, BCR-induced activation of cofilin initiates remodeling of the submembrane actin cytoskeleton. Although the mechanism by which BCR signaling increases the amount of active, non-phosphorylated cofilin is not fully understood, it could include transient inhibition of LIMK. As the B cell spreads, localized activation of LIMK could suppress cofilin activity in nascent lamellipodia so that peripheral actin polymerization can generate stable membrane protrusions. Subsequently, LIMK could tune the level of cofilin activity in order to balance actin network disassembly at the inner face of the peripheral actin ring with actin polymerization at the cell membrane. This actin treadmilling would allow the B cell to continually extend membrane protrusions across the Ag-coated surface. The ability to image the subcellular localization of activated LIMK in real time could provide important insights into the regulation of peripheral actin dynamics. LIMK activity is controlled by the RhoA GTPase and its downstream effector ROCK (Prunier et al., 2017). The BCR activates RhoA (Saci and Carpenter, 2005) but the role of RhoA and ROCK in regulating B cell actin dynamics is not fully understood.

### The Wdr1-LIMK-Cofilin Axis Regulates cSMAC Formation and BCR Signaling at the Immune Synapse

When B cells encounter Ags that are mobile within a membrane, Arp2/3 complex-dependent actin retrograde flow drives the centripetal movement of BCR-Ag microclusters that form at the periphery of B cell-APC contact site (Bolger-Munro et al., 2019). This, together with the progressive coalescence of BCR microclusters, amplifies microcluster-based BCR signaling and promotes cSMAC formation. Arp2/3 complex-dependent peripheral actin dynamics is strongly dependent on concomitant actin disassembly (Carlier et al., 1997; Svitkina and Borisy, 1999). Actin retrograde flow requires the disassembly of aged actin filaments by actin disassembly factors such as cofilin and destrin/ADF (Hotulainen et al., 2005; Delorme et al., 2007; Flynn et al., 2012). Moreover, the inactivation of cofilin decreases the speed of actin retrograde flow (Ohashi et al., 2011; Flynn et al., 2012; Vitriol et al., 2013). We found that depleting cofilin or Wdr1 in B cells substantially reduced the velocity of actin retrograde flow and that this was associated with reduced or delayed cSMAC formation as well as decreased BCR signaling and signal amplification in response to APC-bound Ags. Although LIMK inhibition results in increased cofilin activity, we showed that this also blocked actin retrograde flow, reduced cSMAC formation, and decreased BCR signal amplification. Hence, B cell responses to APC-bound Ags require an optimal level of cofilin activity and are impaired by both deficient and excessive cofilin activity.

This is the first report, to our knowledge, that Wdr1 is important for B cell responses to APC- or membrane-bound Ags. We had previously shown that cofilin-mediated actin severing is important for APC-induced microcluster formation and BCR signaling (Freeman et al., 2011). In that study, we used the phosphomimetic S3D mutant form of cofilin, as well as cell-permeable peptides containing the actin-binding domains of cofilin, both of which competitively inhibit the binding of cofilin to actin filaments and prevent severing (Eibert et al., 2004; Elam et al., 2017; Tanaka et al., 2018). However, these approaches may also interfere with the actin-binding and severing activities of destrin/ADF, which is highly related to cofilin in both its actin-binding domain structure and actin-severing function (Lappalainen et al., 1998; Hotulainen et al., 2005). Both the non-muscle cofilin-1 isoform and destrin are co-expressed in most cell types (Lappalainen et al., 1998). We have shown here that selectively depleting cofilin-1 impairs B cell responses to APC-bound Ags. The contribution of destrin/ADF, and other actin-severing proteins such as gelsolin, to these responses remains to be determined.

Consistent with their cooperative mode of action, depleting either cofilin or Wdr1 had similar effects on B cell responses to APCs. An unexpected finding was that both cofilin depletion and Wdr1 depletion resulted in increased gathering of Ag into clusters at 5 min after the initiation of B cell-APC contact. Actin structures help maintain the integrity of BCR microclusters (Treanor et al., 2011). A reduction in cofilin-mediated disassembly of these actin structures may increase the stability of nascent microclusters and thereby increase the amount of Ag that is gathered into small microclusters during the first 5 min of B cell-APC interactions. Despite the increased Ag clustering, cSMAC formation was delayed in cofilin-depleted B cells and the percent of cells that formed a cSMAC after 15–30 min was consistently lower when cofilin or Wdr1 was depleted. This could be due in part to reduced actin retrograde flow, which would decrease the centripetal movement of BCR-Ag microclusters that form at periphery of the B cell-APC contact site. As well, cortical actin structures that act as diffusion barriers for membrane proteins would be turned over (i.e., disassembled) at a reduced rate in cells that have been depleted of cofilin or Wdr1. The increased lifetime of these actin-based diffusion barriers would limit BCR microcluster mobility within the membrane, resulting in decreased microcluster coalescence and cSMAC formation.

Although the mechanisms are not fully understood, actin retrograde flow amplifies Ag receptor signaling at the T- and B-cell immune synapses (Basu and Huse, 2017; Bolger-Munro et al., 2019). The actin-driven centripetal movement of BCR-Ag microclusters promotes their coalescence into larger clusters (Bolger-Munro et al., 2019) where BCR signaling is enhanced (Liu et al., 2010, 2011; Ketchum et al., 2014). BCR clustering is an essential early amplification event in BCR signaling that is directly related to BCR signaling output (Liu et al., 2010). Recruitment of the Syk tyrosine kinase to Ag-bound BCRs enables Syk-dependent phosphorylation of the CD79 ITAMs via BCR-BCR collisions, allowing those BCRs to recruit Syk and further activate downstream BCR signaling pathways. Microcluster growth leads to further increases in BCR signaling (Liu et al., 2010, 2011; Ketchum et al., 2014). As the microclusters grow in area, a greater number of BCRs in the interior of the cluster may be shielded from inhibitory CD22-SHP1 complexes (Gasparrini et al., 2016). CD22 is a transmembrane protein that limits BCR signaling by recruiting the SHP1, a phosphatase that can terminate BCR signaling by dephosphorylating the CD79 ITAMs. Actin retrograde flow may also amplify BCR signaling by exerting force on the cytoplasmic domains of BCRs that are bound to APC-associated Ags. The BCR is a mechanosensitive receptor and increased mechanical tension on the BCR results in enhanced recruitment of Syk and other signaling components to the BCR (Wan et al., 2013, 2015; Liu et al., 2014; Shaheen et al., 2019).

We showed that depleting either cofilin or Wdr1 significantly reduced the velocity of the actin retrograde flow and that this correlated with a reduction in BCR signal amplification. When either cofilin or Wdr1 was depleted the amount of signaling generated per unit of Ag gathered into BCR-Ag microclusters was significantly reduced during the first 10 min of B cell-APC interaction. A similar decrease in BCR signal amplification is observed when Arp2/3 complex activity is inhibited (Bolger-Munro et al., 2019). This suggests that cofilin and the Arp2/3 complex work in concert to support actin-dependent processes that amplify microcluster-based BCR signaling. The reduced signal amplification in Wdr1- and cofilin-depleted cells contributed to significant decreases in the total amount of pCD79 present in BCR microclusters during the first 10–30 min of B cell-APC interaction. We have previously shown that such decreases in initial APC-induced BCR signaling are associated with impaired B cell activation (Bolger-Munro et al., 2019).

LIMK is a key negative regulator of cofilin that phosphorylates cofilin on S3 and prevents cofilin from binding to actin filaments. LIMK inhibitors have been widely used to investigate the effects of increased cofilin activity. In T cells, inhibiting or depleting LIMK, or its upstream activator ROCK, results in a larger immune synapse area and increased APC-induced Ca^2+^ signaling (Thauland et al., 2017). Thus, in T cells, LIMK normally limits T cell spreading on the APC surface. This suggests that increased cofilin activity enhances T cell immune synapse formation and APC-induced TCR signaling. In contrast, we found that inhibiting LIMK activated cofilin in B cells but reduced B cell spreading and impaired B cell responses to APC-bound Ags. When LIMK was inhibited, the ability of A20 B cells to gather Ags into clusters at the B cell-APC interface was reduced, as was cSMAC formation. Importantly, at early time points after adding B cells to APCs, both pCD79 levels and BCR signal amplification were significantly lower in LIMKi3-treated A20 B cells and primary B cells than in control cells. The reduced cSMAC formation and BCR signal amplification in LIMKi3-treated B cells may be due to the substantially impaired actin retrograde flow in these cells, which could be a consequence of the thinner peripheral actin network that results from excessive cofilin activity. Although LIMK has multiple substrates (Prunier et al., 2017), the similar alterations in actin-dependent B cell responses caused by inhibiting LIMK, depleting cofilin, and depleting Wdr1 suggest that LIMK-mediated regulation of cofilin is essential for the dynamic actin remodeling that optimizes B cell responses to APCs. Importantly, these data show that both reduced and excessive cofilin activity inhibit actin retrograde flow and impair BCR microcluster centralization and signaling at the immune synapse.

### Perspectives

Immune synapse formation allows lymphocytes to establish a polarized contact site with an APC. At this synapse, the actin-dependent centripetal movement and progressive clustering of antigen receptors amplifies their signaling and allows small amounts of APC-bound Ag to trigger lymphocyte activation. In contrast, B cell activation by soluble Ags that are uniformly distributed in the surrounding medium does not involve the formation of polarized structures and is much less dependent on actin reorganization. Consistent with this idea, we found that actin remodeling driven by the Wdr1-cofilin-LIMK axis amplifies BCR signaling in response to membrane-bound Ags but that it is not essential for responses to soluble BCR ligands. Similarly, the Arp2/3 complex (Bolger-Munro et al., 2019) and the Rac/Cdc42 activator DOCK8 (Randall et al., 2009; Sun et al., 2018), both of which are essential for immune synapse formation, are important for B cells to respond to membrane-bound Ags but dispensable for responses to soluble Ags. Together, these findings support the idea that the actin-dependent movement and spatial reorganization of BCRs at the immune synapse has a unique and critical role in APC-induced B cell activation.

Ag presentation by APCs is an important mode of B cell activation *in vivo*, especially for large Ags that cannot freely diffuse into the B-cell follicles within lymphoid organs (Batista and Harwood, 2009; Cyster, 2010; Heesters et al., 2016). In particular, the ability of subcapsular macrophages to capture bacteria and viruses, and then present them to follicular B cells, is important for the generation of protective antibodies that prevent recurring infections (Junt et al., 2007; Gaya et al., 2015; Moran et al., 2019). The actin-dependent amplification of BCR signaling in response to APC-bound Ags may reduce the amount of Ag required to exceed the threshold for triggering B cell activation. Indeed, the increased actin dynamics in B cells that have been exposed to TLR ligands renders B cells more sensitive to small amounts of Ag (Freeman et al., 2015). Conversely, mutations that result in impaired or aberrant actin dynamics may ablate the actin-dependent amplification of BCR signaling at the immune synapse and render B cells unable to respond to low-affinity Ags or Ags that are present at low density on the APC surface. The *in vitro* activation of naïve murine B cells by APC-bound Ags is reduced when the Arp2/3 complex is inhibited, and this is associated with reduced BCR signaling during the first 5 min of B cell-APC interaction (Bolger-Munro et al., 2019). *In vivo*, B cell-specific deletion of WASp in mice impairs antibody responses to low amounts of Ag (Westerberg et al., 2005). Patients with loss-of-function mutations in WASp or DOCK8 exhibit poor vaccine responses although this may be due in large part to the impaired B cell development and maturation (Biggs et al., 2017; Candotti, 2018). Nevertheless, immune disorders linked to mutations in actin-regulatory proteins are commonly associated with recurring infections (Tur-Gracia and Martinez-Quiles, 2021). Because the actions of the Arp2/3 complex and cofilin are intertwined, cofilin and its network of regulators may also control the threshold for APC-dependent B cell activation *in vivo*, impacting the ability to mount antibody responses to pathogens and vaccines. Testing this hypothesis may require the generation of mice with hypomorphic alleles of these actin regulators such that B cell development and maturation are not impaired. Both mice and humans with hypomorphic alleles of the *Wdr1* gene have been described but their B cell responses to immunization or vaccine challenge have not been investigated (Kile et al., 2007; Seppanen, 2018). LIMK inhibitors have been used in mouse models of disease (Prunier et al., 2017). This approach could be used to test the idea that LIMK tunes the threshold for B cell activation *in vivo*.

The magnitude of BCR signaling determines whether Ag encounter results in B cell activation or immunological tolerance (Cashman et al., 2019; Meffre and O'Connor, 2019; Tan et al., 2019). Autoimmunity can result from excessive BCR signaling or from impaired BCR signaling that fails to eliminate self-reactive B cells. During B cell development, immature B cells are exposed to many self-Ags in the bone marrow. If the binding of the self-Ag to the BCR elicits strong signaling, these self-reactive B cells undergo apoptosis and are deleted. Reduced BCR signaling could impair the ability to delete self-reactive B cells. These B cells could then enter the circulation, and under certain conditions, become activated by self-Ags in peripheral tissues, leading to autoimmunity. Although it is not known whether immune synapse formation plays a role in the deletion of B cells that bind cell-associated Ags in the bone marrow, plasma membrane proteins are strong inducers of B cell clonal deletion (Hartley et al., 1991; Ait-Azzouzene et al., 2005; Nemazee, 2017). Mutations that ablate the actin-dependent amplification of APC-induced BCR signaling could lead to a failure of this central tolerance mechanism. Patients with Wdr1 loss-of-function mutations have increased numbers of immature transitional B cells in the periphery, which could be self-reactive B cells that escaped negative selection in the bone marrow (Pfajfer et al., 2018). Indeed, many of the immunodeficiency syndromes that are due to mutations in actin-regulatory proteins are accompanied by autoimmunity and the production of self-reactive antibodies (Sprenkeler et al., 2020). These immune dysregulation syndromes have been termed actinopathies (Sprenkeler et al., 2020). Further work is needed to test the hypothesis that actin-dependent amplification of BCR signaling at the immune synapse tunes the threshold for the deletion of self-reactive B cells in the bone marrow.

## Data Availability Statement

The raw data supporting the conclusions of this article will be made available by the authors, without undue reservation.

## Ethics Statement

The animal study was reviewed and approved by University of British Columbia Animal Care Committee.

## Author Contributions

MB-M, KC, FC, and YL conceived and designed the experiments. MB-M, KC, FC, YL, MD-L, ND, and CK performed the experiments. MB-M, KC, FC, YL, and MG analyzed the results. MB-M, FC, YL, and MG wrote the manuscript with input from KC. MG was the principal investigator of the study. All authors contributed to the article and approved the submitted version.

## Conflict of Interest

The authors declare that the research was conducted in the absence of any commercial or financial relationships that could be construed as a potential conflict of interest.

## Footnotes

^1^https://github.com/mbolgermunro/FIJImacros/blob/master/APC_analyser_MBM.ijm

## Abbreviations

ADF: actin-depolymerizing factor
Ag: antigen
APC: antigen-presenting cell
Arp2/3: actin-related protein 2/3
BCR: B cell antigen receptor
BSA: bovine serum albumin
cSMAC: central supramolecular activation cluster
Cotl1: coactosin-like 1
F-actin: filamentous actin
FCS: fetal calf serum
HEL: hen egg lysozyme
Ig: immunoglobulin
ITAM: immunoreceptor tyrosine-based activation motif
LIMK: LIM domain kinase
LIMKi3: LIMK inhibitor 3
mHBS: modified HEPES-buffered saline
pCD79: phosphorylated CD79
PFA: paraformaldehyde
ROCK: Rho-associated protein kinase
STED: stimulated emission depletion
TIRF: total internal reflection fluorescence
TLR: Toll-like receptor
Wdr1: WD repeat-containing protein 1.
