# Supplementary material for: The Wdr1-LIMK-Cofilin Axis Controls B Cell Antigen Receptor-Induced Actin Remodeling and Signaling at the Immune Synapse
Source: Front Cell Dev Biol. 2021 Apr 13;9:649433. doi: 10.3389/fcell.2021.649433 (PMC8076898; doi:10.3389/fcell.2021.649433)
Supplement: Supplementary file 6 [file Data_Sheet_1.PDF]

Supplementary Material

1. Supplementary Figures 1-6
2. Supplementary Table 1
3. Captions for Supplementary Movies 1-5

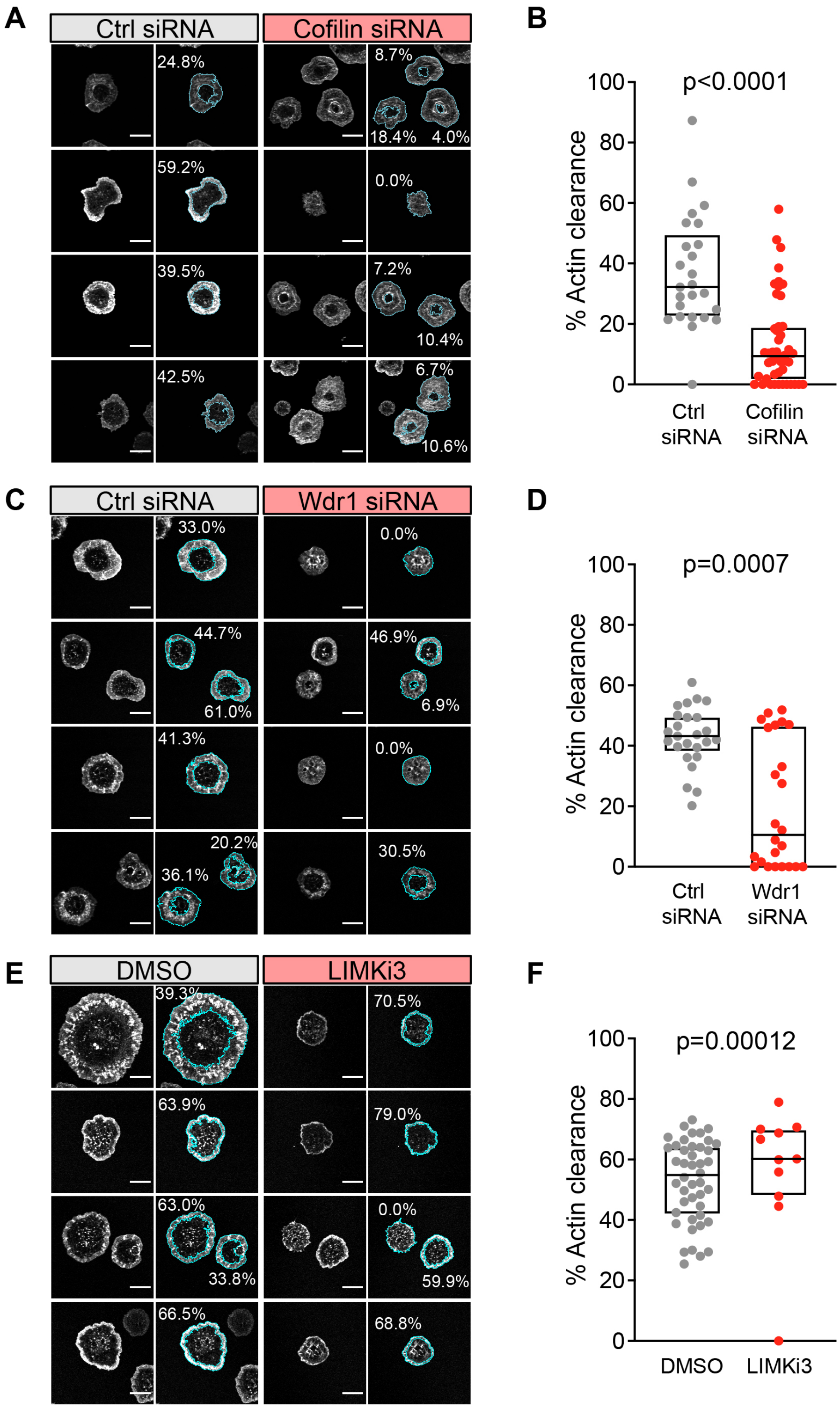

SUPPLEMENTARY FIGURE 1 | Depletion of F-actin from the central region of spreading B cells is regulated by cofilin, Wdr1, and LIMK. A20 B cells were transfected with control (Ctrl) siRNA or cofilin siRNA **(A,B)**, transfected with control (Ctrl) siRNA or Wdr1 siRNA **(C,D)**, or pre-treated with DMSO or 50 µM LIMKi3 for 1 h **(E,F)**. The cells were then allowed

to spread on anti-IgG-coated coverslips for 30 min before being stained with rhodamine-phalloidin and imaged by confocal microscopy. Representative images are shown in panels **A**, **C**, and **E**. The outer edge of the peripheral actin ring was used to calculate the total cell area. The inner face of the peripheral actin ring (blue traces) was used to delimit the central actin-depleted region of the cell and calculate its area, which is expressed as a percent of the total cell area. These values are shown for the representative cells in panels **A**, **C**, and **E**. Where the percent of the cell area that was cleared of actin is indicated as 0%, there was no single contiguous region in which F-actin staining was below the threshold value. The bar graphs in panels **B**, **D**, and **F** show representative experiments in which the percent actin clearance was calculated for >25 cells **(B)**, >15 cells **(D)** or >11 cells **(F)** per treatment group. p-values were determined using the Mann-Whitney U test. **Figures 2C, F, and I** show complied data from multiple experiments.

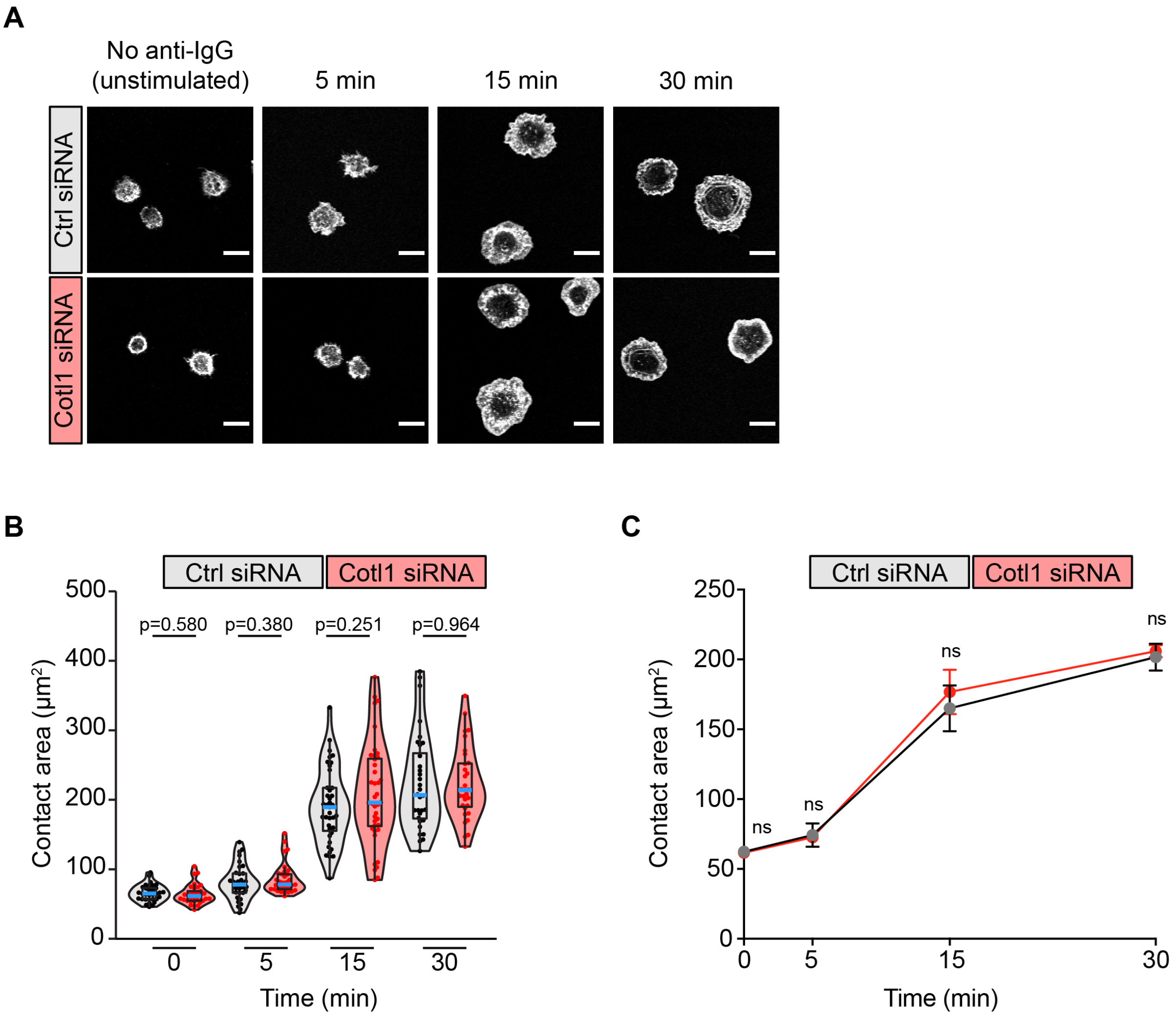

SUPPLEMENTARY FIGURE 2 | Depleting Cotl1 does not alter B-cell spreading on immobilized anti-Ig. A20 B cells were transfected with control siRNA (Ctrl) or Cotl1 siRNA. The cells were then allowed to spread on anti-IgG-coated coverslips for the indicated times before being stained with rhodamine-phalloidin. For the "no anti-IgG (unstimulated)" controls (0 time points on the graphs), the cells were added to bare coverslips for 5 min. **(A)** Representative confocal microscopy images are shown. Scale bars: 10 µm. **(B)** The cell area was quantified using the actin staining to define the cell edge. Each dot in the beeswarm plot represents one cell and the median (blue line) and interquartile ranges (black box) for >30 cells are shown for each time point. Representative data from one of three independent experiments. p-values were determined using the Mann-Whitney U test. **(C)** For each time point, the mean ± SEM for the median values from the three independent experiments is graphed. Two-tailed paired t-tests showed that there were no significant differences (ns, not significant; $p > 0.05$) at any time point.

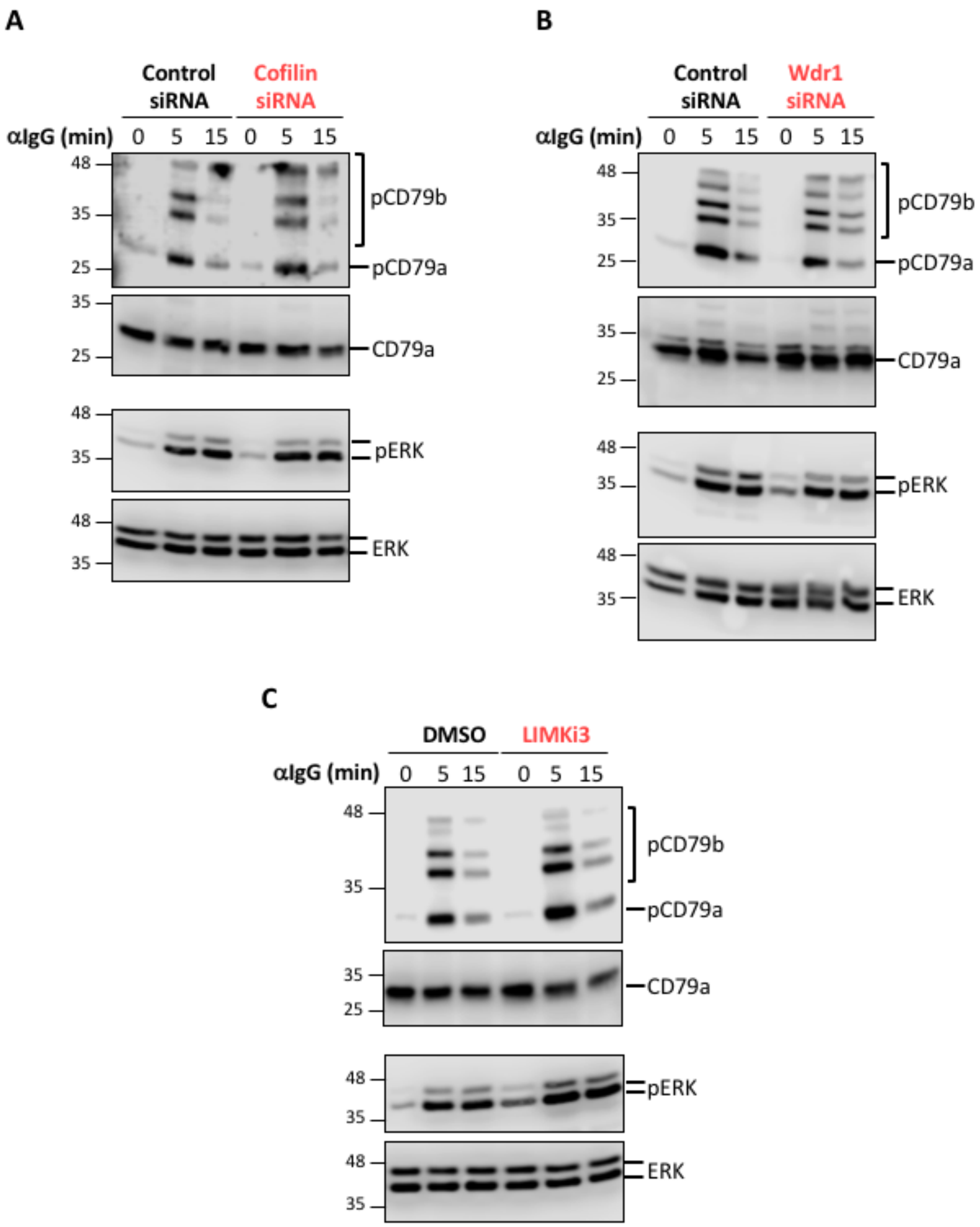

SUPPLEMENTARY FIGURE 3 | Targeting cofilin, Wdr1, or LIMK does not impair initial BCR signaling in response to soluble anti-Ig antibodies. A20 B cells were **(A)** with control siRNA or cofilin siRNA, **(B)** transfected with control siRNA or Wdr1 siRNA, or **(C)** pre-treated with DMSO or 50 µM LIMKi3 for 1 h. The cells were then stimulated with 20 µg/mL goat anti-mouse IgG (αIgG) for the indicated times. Cell extracts were analyzed by immunoblotting with antibodies that recognize the phosphorylated ITAMs in CD79a and CD79b (pCD79a, pCD79b) or the phosphorylated, active form of ERK (pERK). As loading controls, the same cell extracts were probed with antibodies against total CD79a or total ERK. Molecular weight markers (in kDa) are indicated to the left of each blot. For each panel, one of three independent experiments with similar results is shown.

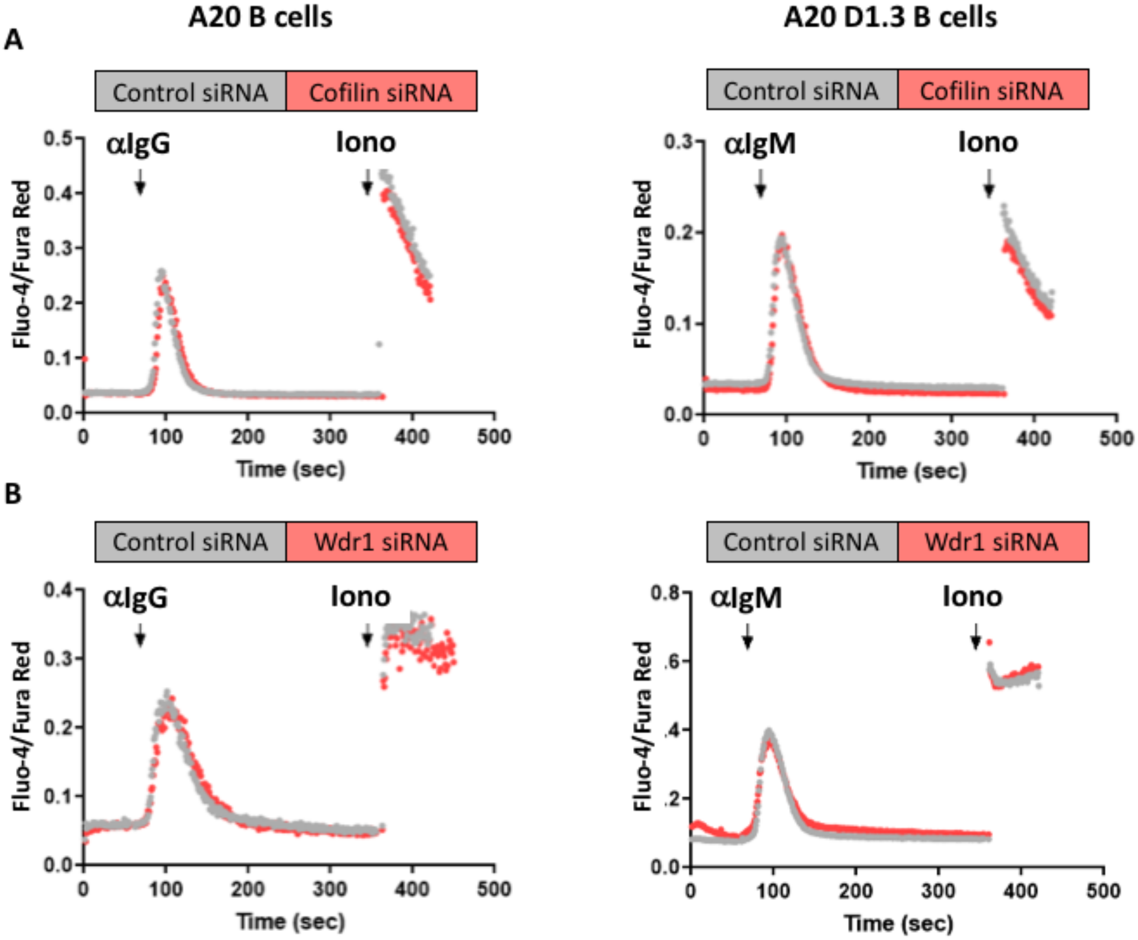

SUPPLEMENTARY FIGURE 4 | Targeting cofilin or Wdr1 does not impair the ability of soluble anti-Ig antibodies to stimulate increases in cytoplasmic Ca^{2+}. A20 B cells (left panels) or A20 D1.3 B cells (right panels) were **(A)** transfected with control siRNA or cofilin siRNA or **(B)** transfected with control siRNA or Wdr1 siRNA. After loading with Ca^{2+}-sensitive dyes, A20 B cells were stimulated with 20 µg/mL goat anti-mouse IgG (αIgG) and A20 D1.3B cells were stimulated with 20 µg/mL goat anti-mouse IgM (αIgM) to engage the D1.3 BCR. Subsequently, ionomycin was added to saturate the dyes and provide an internal standard for comparing samples. For each panel, one of two independent experiments with similar results is shown. Although LIMKi3-treated cells also generated strong Ca^{2+} responses to anti-IgG antibodies, the ionomycin responses were reduced in the LIMKi3-treated cells, preventing a direct comparison between the DMSO- and LIMKi3-treated cells.

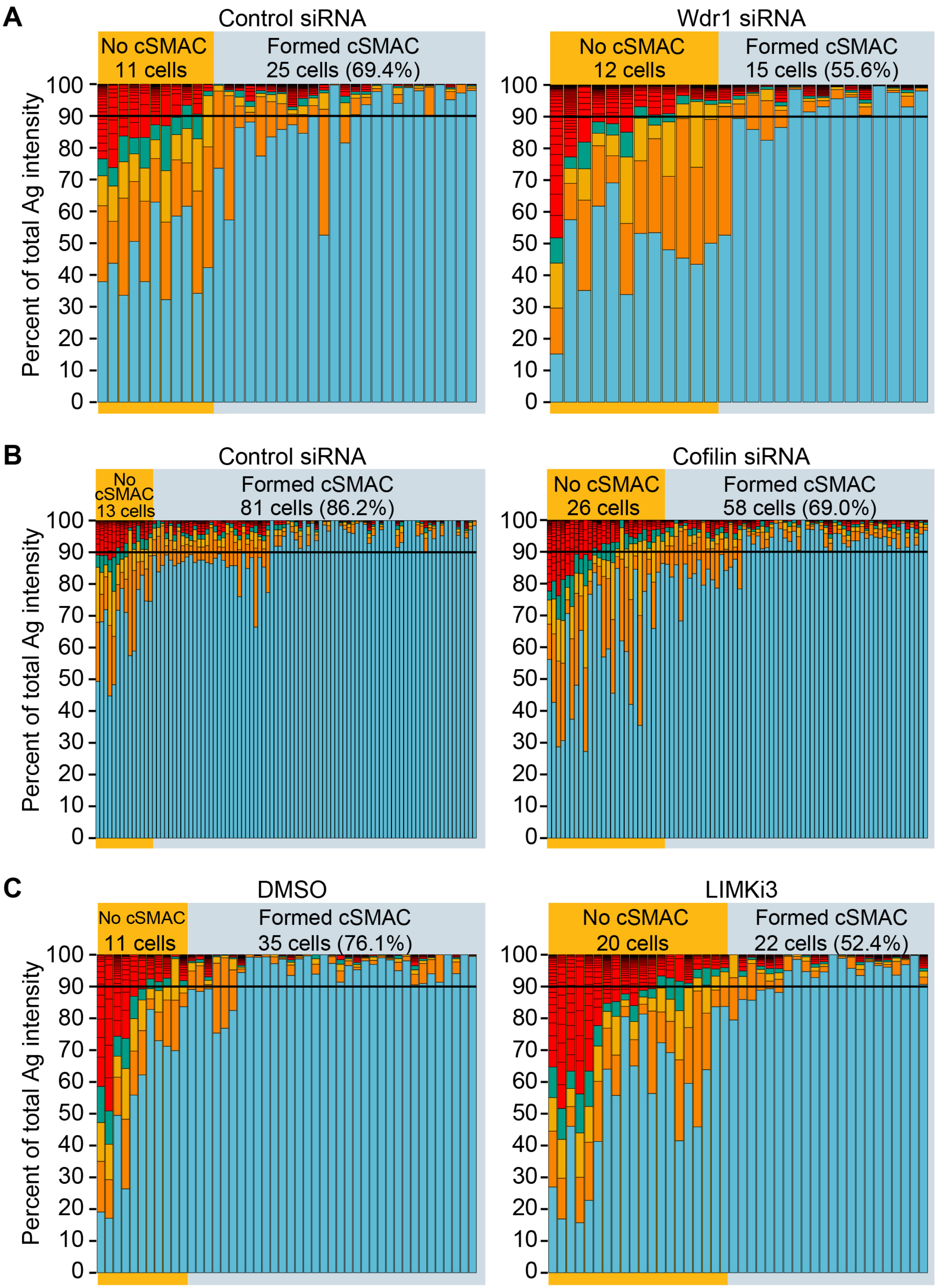

SUPPLEMENTARY FIGURE 5 | Targeting Wdr1, cofilin, or LIMK reduces the percent of cells that form a cSMAC. A20 D1.3 B cells that had been **(A)** transfected with control siRNA or Wdr1 siRNA, **(B)** transfected with control siRNA or cofilin1 siRNA, or **(C)** pre-treated with

DMSO or 50 μM LIMKi3 for 1 h were added to mHEL-HaloTag-expressing COS-7 APCs. The cells were fixed after 30 min and the B cell-APC interface was imaged by spinning disk microscopy. The stacked bar plots show the fraction of the total Ag fluorescence intensity in individual clusters. Each bar represents one cell and each colored segment within a bar represents a single Ag cluster. The size of the colored segment is proportional to the fraction of the cell's total Ag fluorescence intensity that is contained within that cluster. Cells in which >90% of the total Ag fluorescence intensity (black horizontal lines) is contained with 1-2 clusters (i.e. the sum of the blue segment and orange segment of the bar is >90%) were deemed to have formed a cSMAC. Each panel is one of 3-4 independent experiments that are compiled in **Figure 5B** (Wdr1), **Figure 6B** (cofilin), or **Figure 7B** (LIMK).

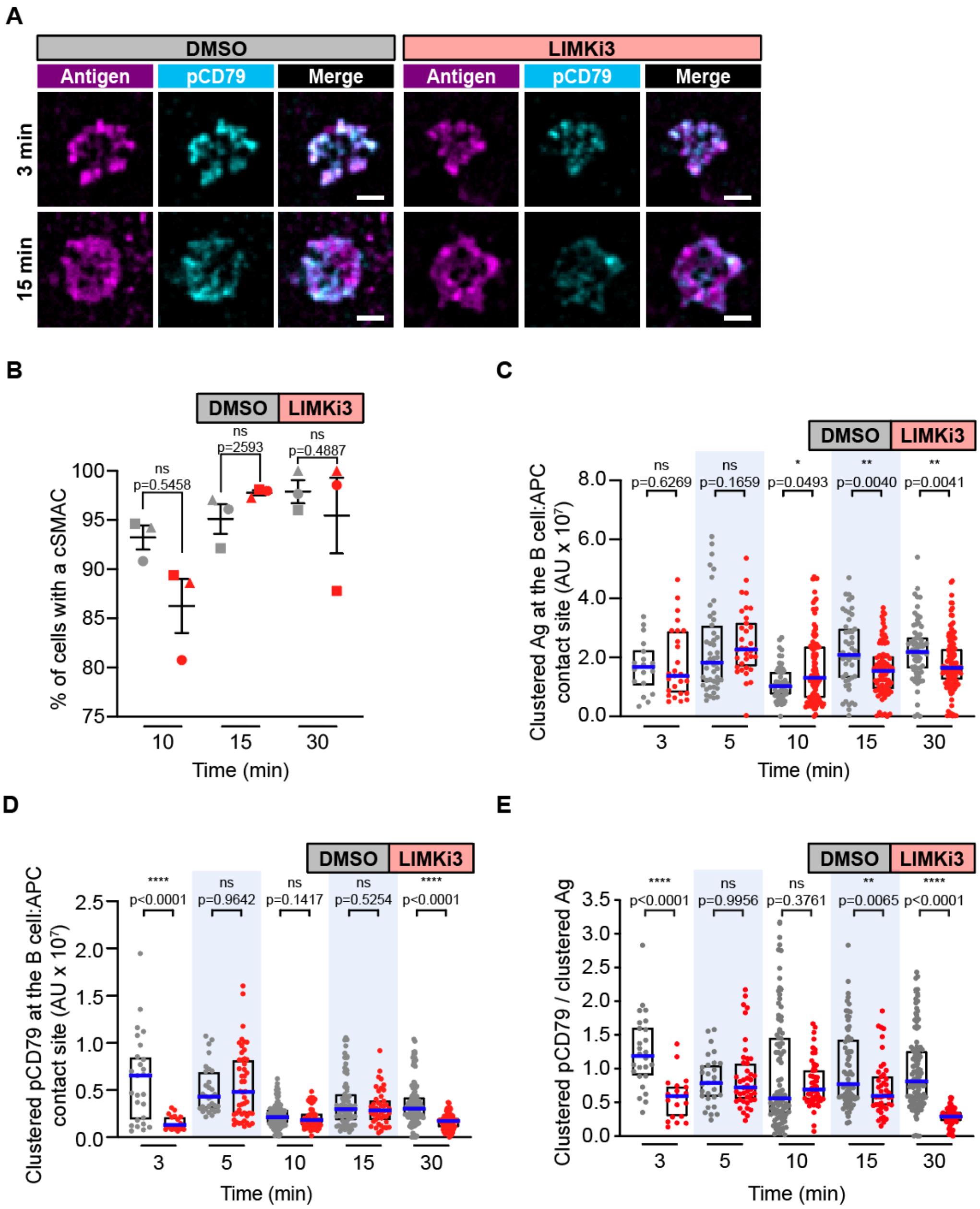

SUPPLEMENTARY FIGURE 6 | Inhibiting LIMK in primary B cells impairs initial BCR signaling at the immune synapse. *Ex vivo* MD4 B cells were pre-treated with DMSO or 1 µM LIMKi3 for 1 h before being added to mHEL-HaloTag-expressing COS-7 APCs. The cells were then fixed at the indicated times and the B cell-APC interface was imaged by spinning disk microscopy. **(A)** Representative images. Scale bars: 5 µm. **(B)** The percent of cells that had formed a cSMAC is graphed. Each symbol is an independent experiment. Paired t-tests were used to calculate p-values. **(C,D)** The total fluorescence intensity of clustered mHEL-HaloTag Ag **(C)** or clustered pCD79 **(D)** at the B cell-APC contact site was quantified for each cell. Each dot is one cell. n >30 cells per condition. The median (blue line) and interquartile ranges (black box) are shown. **(E)** For each B cell in **(C)** and **(D)**, the total fluorescence intensity of clustered pCD79 was divided by the total fluorescence intensity of clustered Ag. The data in panels **C-E** are from the same experiment, which is representative of three independent experiments. The Mann-Whitney U test was used to calculate p-values for panels **C-E**. ****p<0.0001; **p<0.01; *p≤0.05; ns, not significant (p>0.05).

Supplementary Table 1

Cell line/LOF approach	Expt #	Mean Surface IgG (% control)	Mean Surface IgM (% control)	Mean F-actin (% control)	Mean FSC (% control)
**A20/Cofilin siRNA**	1	105.0	-	126.0	99.9
	2	96.0	-	126.5	97.5
	3	131.1	-	111.9	102.2
	**Mean ± SEM**	**110.7 ± 10.5**	**-**	**121.5 ± 4.8**	**99.9 ± 1.4**
	**p-value**	**0.4168**	**-**	**0.046**	**0.9329**

Cell line/LOF approach	Expt #	Mean Surface IgG (% control)	Mean Surface IgM (% control)	Mean F-actin (% control)	Mean FSC (% control)
**A20 D1.3/Cofilin siRNA**	1	101.3	103.3	118.7	92.9
	2	99.8	102.4	103.0	104.0
	3	99.7	112.0	123.9	99.4
	4	171.3	131.9	114.8	104.3
	**Mean ± SEM**	**118.0 ± 17.8**	**112.4 ± 6.9**	**115.1 ± 4.4**	**100.1 ± 2.7**
	**p-value**	**0.3857**	**0.1677**	**0.0426**	**0.9604**

Cell line/LOF approach	Expt #	Mean Surface IgG (% control)	Mean Surface IgM (% control)	Mean F-actin (% control)	Mean FSC (% control)
**A20/WDR1 siRNA**	1	96.7	-	-	-
	2	86.4	-	-	-
	3	-	-	105.5	102.5
	4	-	-	125.3	100.0
	5	-	-	108.2	99.2
	6	-	-	87.5	102.8
	7	-	-	120.8	100.9
	8	-	-	115.9	97.3
	9	-	-	90.7	101.2
	10	-	-	88.4	102.3
	**Mean ± SEM**	**91.6 ± 5.2**	**-**	**105.3 ± 5.3**	**100.8 ± 0.7**
	**p-value**	**0.35**	**-**	**0.3515**	**0.2956**

Cell line/LOF approach	Expt #	Mean Surface IgG (% control)	Mean Surface IgM (% control)	Mean F-actin (% control)	Mean FSC (% control)
**A20 D1.3/WDR1 siRNA**	1	97.5	100.0	101.6	102.2
	2	116.3	95.3	108.0	100.5
	3	99.0	100.3	75.2	96.5
	4	111.1	107.6	123.4	101.7
	5	100.8	95.5	84.1	99.9
	6	-	-	96.4	96.3
	7	-	-	82.0	104.3
	8	-	-	95.3	98.3
	9	-	-	83.3	98.2
	10	-	-	113.1	97.1
	11	-	-	84.3	108.2
	12	-	-	108.4	95.0
	13	-	-	70.1	98.6
	**Mean ± SEM**	**104.9 ± 3.7**	**99.7 ± 2.2**	**94.3 ± 4.4**	**99.7 ± 1.0**
	**p-value**	**0.2533**	**0.9051**	**0.2201**	**0.7998**

(continued on next page)

(Supplementary Table 1 continued)

Cell line/LOF approach	Expt #	Mean Surface IgG (% control)	Mean Surface IgM (% control)	Mean F-actin (% control)	Mean FSC (% control)
**A20/LIMKi3**	1	84.1	-	91.1	98.1
	2	97.6	-	101.4	98.7
	3	100.3	-	104.9	97.4
	4	101.8	-	92.4	91.9
	**Mean ± SEM**	**96.0 ± 4.0**	**-**	**97.5 ± 3.4**	**96.5 ± 1.6**
	**p-value**	**0.3925**	**-**	**0.5052**	**0.115**

**A20 D1.3/LIMKi3**	1	105.4	107.4	104.4	97.5
	2	108.0	104.6	107.6	110.0
	3	121.4	97.9	112.0	103.8
	4	130.0	115.0	96.3	103.5
	**Mean ± SEM**	**116.2 ± 5.8**	**106.2 ± 3.5**	**105.1 ± 3.3**	**103.7 ± 2.5**
	**p-value**	**0.068**	**0.1786**	**0.2232**	**0.2428**

SUPPLEMENTARY TABLE 1 | Effects of targeting the Wdr1-LIMK-cofilin axis on cell surface BCR levels, total F-actin content, and cell size. Cells were transfected with siRNAs and then cultured for 48 h, or treated with DMSO or 50 μM LIMKi3 for 1 h. After fixation, the cells were stained with rhodamine-phalloidin to quantify F-actin or stained with anti-Ig antibodies to quantify cell surface levels of the endogenous IgG-BCR or the transfected IgM D1.3 BCR. Flow cytometry was used to quantify fluorescence and forward scatter was used a relative measure of cell size. The mean fluorescence intensity (MFI) or mean forward scatter is expressed as a percent of the mean value for the corresponding control siRNA-transfected cells or DMSO-treated cells in the same experiment (defined as 100%). For each condition, the mean ± SEM for the indicated number of experiments is reported. p-values were determined using paired two-tailed t-tests. Only the values highlighted in yellow were significantly different ($p < 0.05$) than the values for the control cells.

CAPTIONS FOR SUPPLEMENTARY MOVIES

MOVIE 1 | Peripheral actin dynamics in control siRNA-transfected B cells plated on immobilized anti-IgG. A20 B cells that had been co-transfected with F-tractin-GFP and control siRNA were added to anti-IgG-coated coverslips. The contact site was then imaged using TIRF microscopy. Images were acquired every 1 s for 10 min. Video playback is 60 frames per second (60X real speed). See Figure 4A.

MOVIE 2 | Peripheral actin dynamics in cofilin siRNA-transfected B cells plated on immobilized anti-IgG. A20 B cells that had been co-transfected with F-tractin-GFP and cofilin siRNA were added to anti-IgG-coated coverslips. The contact site was then imaged using TIRF microscopy. Images were acquired every 1 s for 10 min. Video playback is 60 frames per second (60X real speed). See Figure 4A.

MOVIE 3 | Peripheral actin dynamics in Wdr1 siRNA-transfected B cells plated on immobilized anti-IgG. A20 B cells that had been co-transfected with F-tractin-GFP and Wdr1 siRNA were added to anti-IgG-coated coverslips. The contact site was then imaged using TIRF microscopy. Images were acquired every 1 s for 10 min. Video playback is 60 frames per second (60X real speed). See Figure 4A.

MOVIE 4 | Peripheral actin dynamics in DMSO-treated B cells plated on immobilized anti-IgG. A20 B cells that had been transfected with F-tractin-GFP were pre-treated with DMSO for 1 h and then added to anti-IgG-coated coverslips. The contact site was then imaged using TIRF microscopy. Images were acquired every 1 s for 10 min. Video playback is 60 frames per second (60X real speed). See Figure 4B.

MOVIE 5 | Peripheral actin dynamics in LIMKi3-treated B cells plated on immobilized anti-IgG. A20 B cells that had been transfected with F-tractin-GFP were pre-treated with 50 μM LIMKi3 for 1 h and then added to anti-IgG-coated coverslips. The contact site was then imaged using TIRF microscopy. Images were acquired every 1 s for 10 min. Video playback is 60 frames per second (60X real speed). See Figure 4B.
